# Effect of pro-inflammatory cytokine priming and storage temperature of the mesenchymal stromal cell (MSC) secretome on equine articular chondrocytes

**DOI:** 10.3389/fbioe.2023.1204737

**Published:** 2023-08-31

**Authors:** Manon Jammes, Romain Contentin, Fabrice Audigié, Frédéric Cassé, Philippe Galéra

**Affiliations:** ^1^ Normandie University, UNICAEN, BIOTARGEN, Caen, France; ^2^ Unit Under Contract 957 Equine Biomechanics and Locomotor Disorders (USC 957 BPLC), Center of Imaging and Research on Locomotor Affections on Equines (CIRALE), French National Research Institute for Agriculture Food and Environment (INRAE), École Nationale Vétérinaire d’Alfort, Maisons-Alfort, France

**Keywords:** osteoarthritis, mesenchymal stromal cells, secretome, horse, cartilage, cytokine priming

## Abstract

**Context:** Osteoarthritis (OA) is an invalidating articular disease characterized by cartilage degradation and inflammatory events. In horses, OA is associated with up to 60% of lameness and leads to reduced animal welfare along with extensive economic losses; currently, there are no curative therapies to treat OA. The mesenchymal stromal cell (MSC) secretome exhibits anti-inflammatory properties, making it an attractive candidate for improving the management of OA. In this study, we determined the best storage conditions for conditioned media (CMs) and tested whether priming MSCs with cytokines can enhance the properties of the MSC secretome.

**Methods:** First, properties of CMs collected from bone-marrow MSC cultures and stored at −80°C, −20°C, 4°C, 20°C or 37°C were assessed on 3D cultures of equine articular chondrocytes (eACs). Second, we primed MSCs with IL-1β, TNF-α or IFN-γ, and evaluated the MSC transcript levels of immunomodulatory effectors and growth factors. The primed CMs were also harvested for subsequent treatment of eACs, either cultured in monolayers or as 3D cell cultures. Finally, we evaluated the effect of CMs on the proliferation and the phenotype of eACs and the quality of the extracellular matrix of the neosynthesized cartilage.

**Results:** CM storage at −80°C, −20°C, and 4°C improved collagen protein accumulation, cell proliferation and the downregulation of inflammation. The three cytokines chosen for the MSC priming influenced MSC immunomodulator gene expression, although each cytokine led to a different pattern of MSC immunomodulation. The cytokine-primed CM had no major effect on eAC proliferation, with IL-1β and TNF-α slightly increasing collagen (types IIB and I) accumulation in eAC 3D cultures (particularly with the CM derived from MSCs primed with IL-1β), and IFN-γ leading to a marked decrease. IL-1β-primed CMs resulted in increased eAC transcript levels of *MMP1, MMP13* and *HTRA1*, whereas IFNγ-primed CMs decreased the levels of *HTRA1* and *MMP13*.

**Conclusion:** Although the three cytokines differentially affected the expression of immunomodulatory molecules, primed CMs induced a distinct effect on eACs according to the cytokine used for MSC priming. Different mechanisms seemed to be triggered by each priming cytokine, highlighting the need for further investigation. Nevertheless, this study demonstrates the potential of MSC-CMs for improving equine OA management.

## Introduction

Joint arthropathies are the main cause of lameness in equine athletes and lead to substantial financial losses in the equine industry. In 60% of cases (McIlwraith et al., 2012), lameness is due to osteoarthritis (OA) which can be related to aging or an intense athletic career. In humans, this invalidating pathology affects an estimated 300 million people worldwide (Abramoff and Caldera, 2020). OA is mainly characterized by irreversible degradation of the cartilage, even though all articular tissues are altered, and the whole joint is inflamed. As an avascular and non-innervated conjunctive tissue, hyaline articular cartilage softens shocks, protects bones from friction and helps joint mobility. These properties are directly correlated with its composition. The cartilage extracellular matrix (ECM) contains characteristic molecules, such as type IIB collagen and aggrecan, as well as other less specific proteoglycans whose matrix structure and strong osmotic force ensure mechanical integrity and water retention (Goodrich and Nixon, 2006).

To date, treatments of equine and human OA have only aimed to decrease pain, inflammation and delay the later stages of the disease (Contino, 2018; Abramoff and Caldera, 2020). There is currently no real treatment to regenerate lost hyaline cartilage.

Over the past several years, interest has increased regarding the therapeutic potential of mesenchymal stromal cells (MSCs) for the management of OA, even beyond their usefulness in the context of autologous chondrocyte transplantation (ACT) (Cokelaere et al., 2016; Bertoni et al., 2021). MSCs indeed exhibit anti-inflammatory, anti-fibrotic and immunosuppressive properties, and thus can modulate the recipient immune response. Intra-articular (IA) injections of bone marrow (BM)-MSCs limit the progression of imaging signs of OA in an induced equine model of OA (Cokelaere et al., 2016; Bertoni et al., 2021). However, among others limitations, the use of MSCs can trigger local inflammation and decrease the therapeutic efficiency of MSCs (Herberts et al., 2011; Joswig et al., 2017; Bertoni et al., 2019).

To bypass the limitations inherent to cell therapy, several studies have investigated the therapeutic potential of the MSC secretome, because it contains pleiotropic bioactive elements (Jammes et al., 2023). The composition of the secretome depends highly on parental cell type and metabolism. These compounds can be freely present in biological fluids or enclosed in vesicles of different sizes, known as microvesicles or ectosomes (100–1,000 nm) and exosomes (30–200 nm), collectively known under the term “extracellular vesicles” (EVs). Similar to whole-cell MSCs, anti-inflammatory and regenerative capacities have been attributed to the MSC secretome (Kumar et al., 2019; Hade et al., 2021). Some studies have already demonstrated the potential of MSC-derived secretome in cartilage regeneration and, consequently, their usefulness for OA therapy (Cosenza et al., 2017; Velot et al., 2021). Recently, we showed that an unstimulated BM-MSC secretome promotes collagen production in equine articular chondrocytes (eACs) and increases their migratory capacities (Contentin et al., 2022). Interestingly, intra-articular injections of the MSC secretome in an equine model of joint inflammation show anti-inflammatory effects comparable to those observed with the direct injection of MSCs (Kearney et al., 2022). However, low production yields and lack of disease specificity are limitations that prevent the use of MSC secretome-based therapy to its full capacity (Chen et al., 2022). Furthermore, when eACs are cultured with the naïve MSC secretome, the neosynthesized ECM contains type I collagen, suggestive of fibrocartilage formation (Contentin et al., 2022). Because the MSC-secretome composition highly depends on the environment that the MSCs develop in, it is possible to enhance the secretion of MSCs for therapeutic applications thanks to several methods (Jammes et al., 2023). A pathological situation such as OA triggers the reaction of the immune system and the secretion of pro-inflammatory factors in macrophages and other immune cells recruited to the inflammation site (Rasmusson, 2006). Thus, using pro-inflammatory cytokines to boost MSC therapeutic properties seems to be a logical approach. The main pro-inflammatory cytokine used in most studies for MSC stimulation—also known as preconditioning or priming—is interferon-γ (IFN-γ). Considered as the “gold standard”, IFN-γ stimulates MSC immunosuppressive properties that result in increased secretome-mediated chondroprotective capacities (Maumus et al., 2016; Cassano et al., 2018). In addition, MSCs stimulated with this cytokine show increased potential for reducing collagenase-induced cartilage degradation (Maumus et al., 2016). However, other pro-inflammatory cytokines may also be promising candidates to prime MSCs, such as tumor necrosis factor-α (TNF-α) and interleukin-1β (IL-1β). These two cytokines play a predominant role in OA, and are known to induce the expression of matrix metalloproteinase 13 (MMP13), a disintegrin and metalloproteinase with thrombospondin motif 5 (ADAMTS5) and other matrix metalloproteinases linked to cartilage degradation (Tetlow et al., 2001; Demoor et al., 2014; Zhao et al., 2019). There is substantial evidence of TNF-α efficacy in MSC priming. Alone (Voskamp et al., 2020) or in combination with other pro-inflammatory molecules (Barrachina et al., 2017a; Caffi et al., 2020), this cytokine increases the MSC production of immunomodulatory factors. The decrease in MMP activity induced by TNF-α priming also proves the chondroprotective potential of this cytokine (Giannasi et al., 2020). Likewise, IL-1β priming can improve the anti-inflammatory capacities of MSC secretome (Kim et al., 2021). Recently, the combination of IL-1β and TNF-α showed the ability to enhance human MSC responsiveness to IFN-γ signaling (Hackel et al., 2021). Hence, IFN-γ, IL-1β, and TNF-α are promising candidates to improve the anti-inflammatory properties of the MSC secretome. However, it is still unclear whether the secretome derived from MSCs primed with IFN-γ, IL-1β or TNF-α favors hyaline cartilage synthesis.

Optimizing the therapeutic potential of the MSC-derived secretome through the modulation of the MSC environment is also related to key parameters in preparation protocols. The secretome collected through medium conditioning is typically stored at −80°C; however, many studies have shown that this storage temperature leads to the aggregation of the EVs contained in the conditioned medium (CM), reducing their concentration and affecting their functionality (Jeyaram and Jay, 2017; Gelibter et al., 2022). The choice of storage method is capital for the maintenance of secretome integrity and for the preservation of its therapeutic effects. Thus, the lack of a standardized method hampers the optimization of the investigation into secretome capacities. Although −80°C storage is common, improving CM storage conditions may play a critical role in secretome stability and facilitate the application of the MSC secretome for therapeutic purposes. In 2020, Laggner et al. investigated the impact of different storage conditions (time, temperature, humidity) on various lyophilized secretome parameters, including functionality (Laggner et al., 2020). Nevertheless, no research has been performed on the influence of storage conditions on unprocessed CMs.

The first goal of this study was to determine the best temperature for storing the MSC secretome. To do so, BM-MSC CMs were collected and stored at five classic storage temperatures, namely, −80°C, −20°C, 4°C, room temperature (20°C) and 37°C. Then, we assessed the effect of the CM on eAC viability, proliferation and on the ECM quality of eAC 3D cultures. Furthermore, to enhance the demonstrated effects of the MSC-CM (Contentin et al., 2022), we investigated whether the secretome derived from primed MSCs favors the synthesis of hyaline cartilage ECM. We hypothesized that priming BM-MSCs with pro-inflammatory cytokines helps favor hyaline cartilage ECM. Thus, we primed BM-MSCs with three cytokines—IL-1β, TNF-α or IFN-γ—before conditioning and collecting the CM. Then, the effect of the CM derived from primed MSCs was assessed on eAC viability, proliferation and on the quality of eAC 3D cultures.

## Materials and methods

### Cell isolation and culture

MSCs were isolated from the BM of the sternum of young horses (3–5 years), as previously described (Branly et al., 2017; Branly et al., 2018; Contentin et al., 2020). Briefly, mononucleated cells were collected using density gradient centrifugation and seeded in an isolation medium (Low Glucose-Dulbecco’s modified Eagle Medium (LG-DMEM; Invitrogen; Carlsbad, CA, United States) containing 30% fetal bovine serum (FBS, Invitrogen Life Technologies; Carlsbad, CA, United States), 10^–7^ M dexamethasone (Sigma-Aldrich; Saint-Louis, MO, United States) in a 5% CO_2_ and 95% humidity atmosphere. After colonies appeared, MSCs were expanded and then characterized by their ability to adhere to plastic, to differentiate into osteoblasts, adipocytes and chondrocytes. Furthermore, the expression of characteristic cell surface markers such as CD29, CD44, CD90, and CD105 was checked by flow cytometry (Cytoflex S Flow Cytometer 149, Beckman Coulter France SAS; Paris, France), as was the absence of CD45 and MHC-II expression. The BM-MSCs used in this study were isolated, amplified and characterized as described in our previous works (Branly et al., 2017; Contentin et al., 2020).

eACs were isolated from cartilage biopsies collected from the hock, fetlock or pastern of healthy horses (aged 1–10 years), as previously described (Rakic et al., 2017). Briefly, protease from *Streptomyces griseus* type XIV (Sigma-Aldrich, Saint Louis, MO, United States) was used at 2 mg/mL for 45 min at 37°C for the nonspecific hydrolysis of ECM proteins. Then, collagenase digestion was carried out on small pieces of cartilage for 18 h at 37°C with high glucose-DMEM (HG-DMEM) containing 2 mg/mL *Clostridium histolyticum* type I collagenase (Invitrogen Life Technologies; Carlsbad, CA, United States). Collagenase was inhibited by adding eAC amplification medium (HG-DMEM supplemented with antimicrobial agents (100 IU/mL penicillin, 10 mg/mL streptomycin and 0.025 mg/mL amphotericin B (PSA), Eurobio Scientific; Les Ulis, France) and 10% FBS) and the cell suspension was filtered through a 70 µm nylon strainer. After 300 g centrifugation, cells were seeded at 2 × 10^4^ cells/cm^2^ in eAC amplification medium or frozen in liquid nitrogen (P0 stage). For cell passaging, cells were rinsed twice with 0.01 M phosphate-buffered saline (PBS, Eurobio Scientific, Courtaboeuf, France), trypsinized and seeded at 2 × 10^4^ cells/cm^2^ in eAC amplification medium for further amplification. All strains were tested for the absence of *mycoplasma* contamination using a polymerase chain reaction assay (PCR).

All the methods and procedures described in the present study were approved by the ethics committee “Comité d’éthique/Agence nationale de sécurité sanitaire/École nationale vétérinaire d’Alfort/Université Paris-est Créteil” (ComEth Anses/ENVA/UPEC) (Date of approval: 10 March 2015, permit number: 10/03/15-12).

### Priming and preparation of conditioned media

To maximize the therapeutic potential of the MSC-derived secretome highlighted in our previous study (Contentin et al., 2022), three equine cytokines—IL-1β, TNF-α and IFN-γ—were chosen to stimulate BM-MSC immunomodulatory properties. When BM-MSCs reached 70% confluency, naive cells were rinsed twice with PBS and incubated at 37°C in a 5% CO_2_ atmosphere for 24 h with preconditioning medium, corresponding to LG-DMEM supplemented with antimicrobial agents (100 IU/mL penicillin, 100 μg/mL streptomycin, and 0.25 μg/mL amphotericin B (PSA), Eurobio Scientific; Les Ulis, France) and 20% exosome-depleted FBS (exo-free FBS, Gibco; Grand Island, NY, United States). For priming experiments, equine IL-1β (10 and 20 ng/mL), TNF-α (10 and 20 ng/mL) or IFN-γ (50 and 100 ng/mL) (R&D Systems, Bio-Techne; Minneapolis, Minnesota, United States) were added to the preconditioning medium for 24 h. Preconditioned medium with no cytokine supplementation was also used to obtain naive CM. Then, cells were rinsed twice with PBS and HG-DMEM supplemented with PSA and 2% exo-free FBS (3D culture medium (M3D)) was added for conditioning. In parallel, supplementary M3D was filtered at 0.22 µm, aliquoted and stored at −80°C to use as a control. BM-MSCs were incubated during 24 or 48 h with M3D, and CM were collected, filtered at 0.22 µm, aliquoted and stored at −80 °C (Supplementary Figure S1). To avoid the degradation of secretome components, CMs were only thawed once and discarded if not used.

The procedure used for CM storage experiments was the same as for priming experiments. When 70% confluency was reached, BM-MSCs were rinsed with PBS and incubated for 24 h with preconditioning medium. Cells were then washed twice with PBS and cultured for 24 h with M3D. CMs were filtered at 0.22 µm, aliquoted and stored at −80°C, −20°C, 4°C, room temperature (RT or 20°C) or 37°C for 2 days, 2 weeks or 1 month prior to eAC treatment.

### Three-dimensional culture

Three-dimensional (3D) culture was performed with eACs (passage 2, P2) seeded in bovine type I/III collagen sponges (diameter: 5 mm; thickness: 2 mm) manufactured by Symatèse Biomatériaux as previously described (Rakic et al., 2017). Briefly, each sponge was seeded with 8 × 10^5^ cells and incubated at 37°C in hypoxia (3% O_2_ atmosphere) with M3D (control) or CM. D0 condition corresponds to eAC seeded in the collagen sponges (3D) and cultured for 17 h with the M3D (1 mL/sponge). Depending on the experiments, bone morphogenetic protein 2 (BMP2; dibotermine α, Inductos^®^, Medtronic France SAS, Boulogne-Billancourt, France) chondrogenic molecule was added to M3D or CM at 50 ng/mL. A chondrogenic control was also added to some of the presented experiments (M3D + BMP2 condition). Culture media were changed twice a week (1 mL/sponge), and after 14 days, sponges were rinsed twice with PBS and stored at −80°C.

### Proliferation assay

**FIGURE 1:**
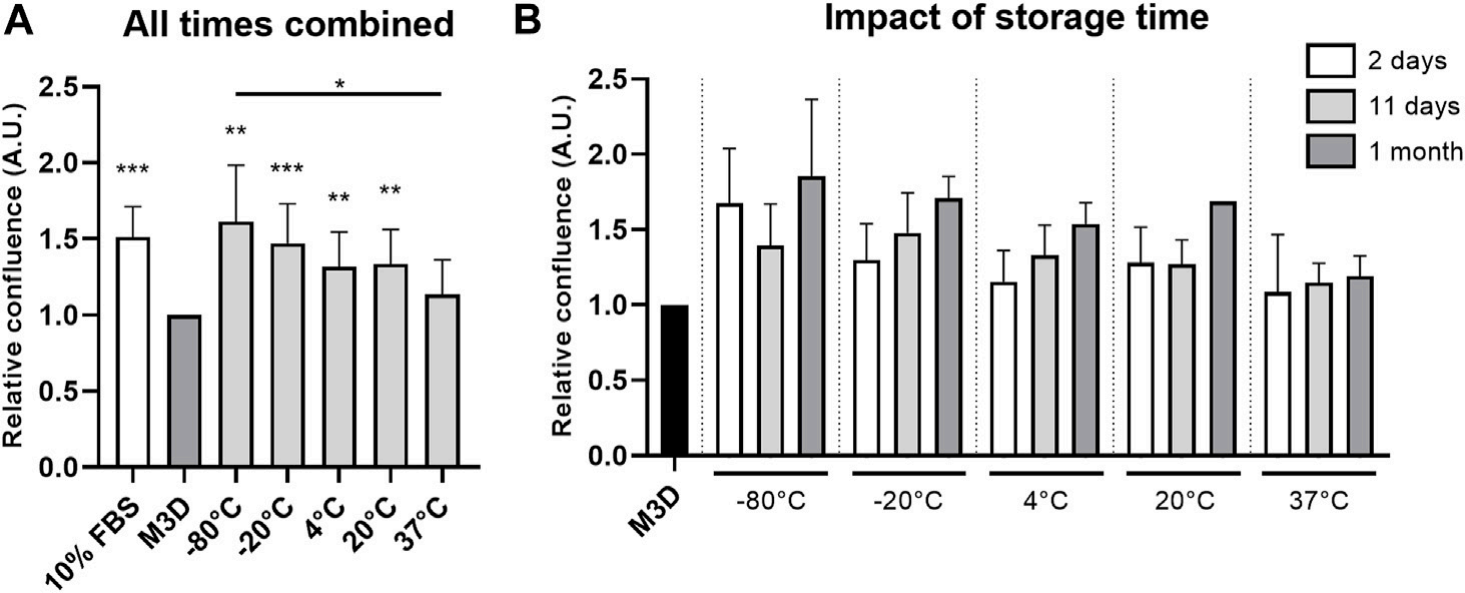
Storage time and temperature affect the CM pro-proliferative effect. CMs from BM-MSCs (P3) were harvested, filtered, aliquoted and stored at different temperatures for 2 days (n = 3), 11 days (n = 3) and 1 month (n = 2). eACs (P2) were seeded in monolayer at 20,000 cells/cm^2^. After 17 h, the culture medium was removed, cells were washed with PBS and treated with CMs. Cultures were monitored for 48 h with an Incucyte^®^ live imaging system. Each condition was tested in triplicate and experiments were repeated 2 or 3 times. Cells were counted on 3 representative areas of each picture taken after 48 h of treatment and just after addition of CM. Relative cell confluence was determined from the ratio of the number of cells at 48 h to the number of cells at 0 h. Histograms show the impact of CM storage temperature—−80°C; −20°C; 4°C; 20°C; 37°C–over all experiments, independently of the storage time (up to 1 month) **(A)**. Impact of each storage time on eAC proliferation rate was also analyzed **(B)**. Mean values are represented as histograms ± standard deviation and analyzed using the Mann-Whitney test; *p < 0.05, **p < 0.01, ***p < 0.005 significantly different from the control M3D condition. 10% FBS: chondrocyte amplification medium; used as positive control; A.U.: arbitrary unit; BM-MSCs: bone marrow-mesenchymal stromal cells; CM: conditioned medium; eACs: equine articular chondrocytes; FBS: fetal bovine serum; M3D: control medium with 2% FBS; P2: passage 2; P3: passage 3.

A 96-well plate was seeded with eACs (P2) at 2 × 10^4^ cells/cm^2^ in eAC amplification medium. After 16 h of culture in normoxia, cells were washed with PBS and 200 µL of treatments (corresponding to the conditions tested, i.e., the control medium and the CMs differentially stored (Figure 1) or the CMs primed or not (Figure 7)) were added to each well. The plate was placed at 37°C in normoxia and monitored for 7 days with the live imaging IncuCyte^®^ technology (IncuCyte S3 microscope and IncuCyte 2021A software, Sartorius; Göttingen, Germany) to assess proliferation rate and cell morphology. Using ImageJ software (ImageJ 1.35c, Wayne Rasband, National Institutes of Health, Bethesda, MD, United States), cells were counted on 3 representative areas of each picture taken after CM addition (200 µL/well) and after 48 h of treatment. The proliferation factor was determined by calculating the ratio between cell number at 48 h and cell number at 0 h. Each condition was tested in triplicate and experiments were reproduced 3 (storage assays) to 5 times (priming assays), depending on the experiment.

### RNA extraction and RT-qPCR

Cells stored at −80°C were thawed and RNA-Solv^®^ (Ozyme; Saint Cyr l’Ecole, France) was used to extract total RNA according to the manufacturer’s instructions. For each sample, 1 µg of total RNA was retro-transcribed into cDNA with the iScript™ Reverse Transcription Supermix (Bio-Rad; Hercules, CA, United States of America) and diluted to 1:20 in nuclease-free water. Real-time PCR was performed with the Go Taq^®^ Probe qPCR Master Mix (Promega; Charbonnières, France) on a CFX96 PCR detection system (Bio-Rad). All primer sequences are listed in Tables 1, 2. The results were normalized using the 2^−ΔΔCT^ method in Bio-Rad CFX Manager software. Each sample was normalized using two housekeeping genes, *β-ACTIN* and *PPIA*.

**TABLE 1:** Primer sequences for MSC gene expression study.

Target gene	Forward sequence	Reverse sequence
*β2M*	CTA CTC TCC CTG ACT GGC CT	TCT GAC CGG TCG ACT TIC ATC
*β-ACTIN*	GAT GAT GAT ATC GCC GCG CTC	TGC CCC ACG TAT GAG TCC TT
*BMP2*	CCT TCG GCC AGA GCT TIT TC	ACG CTA GAA GAC AGT GGG TC
*CD40*	AAG GCC TGG TGG MC TAC AG	TCC TTG GCC ACC TTT CTG ATA C
*CD80*	CAG GAA AGT TGG CTC TGA CCA	TCT CCA TTG TGA TCC TGG CTC
*CD86*	AGT ATA AAG GCC GCA CAA GC	CCT TGG GTA GAT GAG CAG GT
*FGF2*	AGC GAC CCT CAC ATC AAA CT	TCG TIT CAG GGC CAC ATA CC
*HGF*	CCA CAT GAG CAC AGC TAT CG	ACT TTC CCC A7 GCA GGT CA
*IDO1*	TCA TGA CTA CGT GGA CCC AAA A	CGC CTT CAT AGA GCA GAC CTT C
*IGF1*	CTG GAG ATG TAC TGC GCA CC	TGT TTC CCG CAC TCC CTC TA
*IL-6*	CAC CAC TGC AGA CAA AAC CA	CAG GTC TCC TGA TTG AAC CCA
*IL-10*	GAC TTT AAG GGT TAC CTG GGT T	TGC TCT TAT TIT CAC AGG GCA G
*INOS*	GCT GGT CCC CCG ATT TTC TG	AGC ACG GCT TTA ACC AAG AC
*KGF*	AGG CM AGT CAA AGG GAC TCA	TCG TTG CAT TCT TIC TTT GCA T
*P65*	TCC GGA CAC CTC CCT ACG CG	TCG CTG AGC TCC CGA TCC GA
*PPIA*	CCC TAC CGT GTT C7 CGA CA	GTG AAG TCA CCA CCC TGA CA
*TGFβ1*	CCG AGC TCT GGA CAC CAA CTA C	TGC CGT ACG CAG CAG TTC
*TNFAIP6*	GCT TTG TGG GAA GAT ACT GTG G	ACT GGG ITT GGA TGG GGG AT

**TABLE 2:** Primer sequences for eAC gene expression study.

Target gene	Forward sequence	Reverse sequence
*ACAN*	TGT CAA CAA CAA TGC CCA AGA C	CU CU CCG CCC AAA GGT CC
*ADAMTS5*	AAG GGA CAC CAT GTG GCAA A	CCC ACA TGA GCG AGA ACA CT
*β-ACTIN*	GAT GAT GAT ATC GCC GCG CTC	TGC CCC ACG TAT GAG TCC TT
*COL10A1*	GCA CCC CAG TAA TGT ACA CCT ATG	GAG CCA CAC CTG GTC AU TTC
*COL1A1*	TGC CGT GAC CTC AAG ATG TG	CGT CTC CAT GU GCA GM GA
*COL1A2*	CCA GAG TGG AGC AGC GGT TA	GGG ATG UT TCA GGT TGA GCC
*COL2A1*	GGC AAT AGC AGG TTC ACG TAC A	CGA TAA CAG TCT TGC CCC ACT T
*HTRA1*	GGA CU CAT GTT TCC CTC AA	GU CTG CTG AAC AAG CAA CA
*MMP1*	CGA AGG GAA CCC TCG GTG GGA	TGG CCT GGT CCA CAT CTG CTC
*MMP13*	TGA AGA CCC GAA CCC TAA ACA T	GAA GAC TGG TGA TGG CAT CAA G
*P53*	CAC CTG AGG TTG GCT CTG AC	GCA CAA ACA CGC ACC TCA AA
*P65*	CAC GGA TAC CAC CAA GAC CC	GTC TGG ATG CGC TGA CTG AT
*PPIA*	CCC TAC CGT GU CU CGA CA	GTG AAG TCA CCA CCC TGA CA
*PRG4*	CTA CCA CCC AAC GCA ACA AA	ACT GU GTC TCC TTA TTG GGT

### Protein extraction and western blots

Collagen sponges were crushed at 4°C in lysis buffer (RIPA-lysis buffer (50 nM Tris-HCl, 150 mM NaCl, 1 mM NaF, 1 mM egtazic acid (EGTA), 0.25% Na-deoxycholate, 1% NP-40) supplemented just before use with protease and phosphatase inhibitors (phenyl methyl sulfonyl fluoride (1M), pepstatin A (1 μg/mL), aprotinin (1 μg/mL), leupeptin (1 μg/mL) and NA_3_VO_4_ (1 mM)), and were vortexed regularly for 45 min. After a centrifugation step (30 min at 13,000 g), supernatants were collected, and total protein extracts were quantified using the Bradford colorimetric assay (Protein Assay Dye Reagent, Bio-Rad) according to the manufacturer’s instructions. Then, 8–15 µg of total proteins were separated on 7.5% acrylamide gels (TGX Stain-Free Fast Cast Acrylamide Kit 7.5%, Bio-Rad) and transferred to a polyvinylidene difluoride membrane (PVDF, Trans-Blot Turbo RTA Midi PVDF Transfer Kit, Bio-Rad) using a transfer system (Trans-blot^®^ Turbo™, Bio-Rad). Nonspecific sites were blocked with incubation (1 h) at RT with 10% nonfat milk solution prepared in Tris-buffered saline with 0.1% Tween (TBS-T). Membranes were washed 3 times with TBS-T and incubated overnight with antibodies diluted in TBS-T. Characteristics and dilutions of all antibodies are described in Table 3. The following day, membranes were washed three times for 5 min under agitation and incubated with horseradish peroxidase (HRP)-conjugated goat anti-rabbit or goat anti-mouse IgG antibodies (Jackson Immunoresearch; Cambridge, United Kingdom) for 1 h. After three washes of 5 min with TBS-T, membranes were incubated for 5 min in an enhanced chemiluminescent reagent (Clarity Western ECL substrate, Bio-Rad) and proteins were visualized using an imager (ChemiDoc™ Touch Imaging System, Bio-Rad). The quantification of the intensity of each blot was performed using the Image Lab Software (Biorad; Hercules, CA, United States) and normalized to the GAPDH.

**TABLE 3:** Antibodies used for eAC phenotype characterization by Western blot.

Antibody	Dilution	Supplier	References
Rabbit anti-bovine type I collagen	1:3,000	Novotec; Bron, France	20,121–1
Rabbit anti-Human type II collagen	1:750		20,211–1
Mouse anti-Human type X collagen	1:1,000	Sigma-Aldrich; Saint Louis, MO, United States	C7974-2 ML
Rabbit anti-Human GAPDH	1:3,000	Santa Cruz Biotechnology; Dallas, TX, United States	SC-47724
Mouse anti-Human Pcna	1:1,000		SC-56
Rabbit anti-Human HtrA1	1:1,000	Merck Millipore; Billerica, MA, United States	AP1331A
	1:3,000		AP1331B
Rabbit anti-Human type IIB collagen	1:750	Covalab; Villeurbanne, France	-
HRP-conjugated goat anti-rabbit antibody	1:5,000	Jackson Immunoresearch; West Grove, PA, United States	111-035-144
HRP-conjugated goat anti-mouse IgG antibody	1:5,000		115-035-003

### Cytokine dosage

To determine the concentrations of 23 immunomodulatory cytokines in CMs, an equine-specific immunology multiplex assay was carried out (MILLIPLEX^®^ Equine Cytokine/Chemokine Magnetic Bead Panel, Millipore, Merck KGaA; Darmstadt, Germany). This assay included fibroblast growth factor 2 (FGF-2), eotaxin, granulocyte colony-forming stimulating factor (G-CSF), IL-1α, granulocyte-macrophage (GM)-CSF, CX3CL1, IL-13, IL-5, IL-18, IL-1β, IL-6, IL-17A, IL-2, IL-4, IL-12, IFN-γ, IL-8, IFNγ-induced protein 10 (IP-10), CXCL1, CCL2, IL-10, TNF-α and regulated upon activation, normal T cell expressed and presumably secreted (CCL5). A recapitulative table of these molecules is available in the (Supplementary Table S1). Based on the Luminex^®^ xMAP^®^ technology, this assay corresponds to color-coded magnetic beads (MagPlex®-C microspheres) coated with capture antibodies targeting these 23 equine cytokines. The dosages of cytokines were performed on CMs after 24 h of conditioning, and on four different strains. Following the manufacturer’s guidelines, quality control and standards were used to confirm the good execution of the assay and to ensure batch-to-batch consistency. The 96-well plate included in the kit was washed with buffer and 25 µL of standards, controls or non-diluted CMs (prepared and stored at −80°C as previously described) were added in duplicates to the wells. Then, 25 µL of assay buffer, matrix solution and beads were added to each well following the manufacturer’s guidelines and the plate was sealed, placed on an agitator and incubated overnight at 2°C–8°C. After three washes, detection antibodies were added to the wells and incubated for another hour at room temperature. Streptavidin-phycoerythrin was then added to each well, incubated for 30 min at RT and the plate was washed three times before adding sheath fluid. Following the manufacturer’s instructions, measurements were performed with the Luminex^®^ MAGPIX^®^ CCD imager (Luminex Corp.; Texas, United States) and processed with Luminex^®^ xPONENT software. A null value corresponds to a measurement value lower than the detection threshold, specific to each cytokine.

### Statistical analysis

Each experiment was repeated at least three times with different MSC and eAC strains. The Shapiro, Wilk test was used to test the normal distribution of the data. Significance of the results was tested using the Mann-Whitney test. To compare more than two groups we used the Kruskal-Wallis and the Dunn’s tests. The GraphPad Prism 8 software (GraphPad Software Inc.; San Diego, CA, United States) was used to process data and assemble graphs. A *p*-value ≤0.05 was considered significant.

## Results

### Storage time and temperature of MSC-derived CMs modulate eAC proliferation and gene expression

The first aim of this study was to determine whether storage conditions of MSC-derived CMs (MSC-CMs) can be improved to preserve their therapeutic effects with the maximum efficacy. To this end, the impact of three storage durations—2 days, 11 days and 1 month—on MSC-CM properties was assessed at five temperatures that are commonly encountered in biological product conservation protocols, namely, −80°C, −20°C, 4°C, 20°C, and 37°C. We counted the number of cells at the beginning and 48 h after CM treatment to determine the relative confluence (Figure 1, Supplementary Figure S2). Regardless of the storage duration, the eACs cultured with the MSC-CMs stored at 20°C or below had a higher proliferation rate than the eACs cultured with the control medium M3D (Figure 1A, Supplementary Figure S2). Furthermore, the ability of the MSC-CMs to enhance eAC proliferation appeared to decrease with an increase in storage temperature. The maximum proliferation rate was observed when eACs were cultured with CMs stored at negative temperatures, but at higher temperatures, eAC relative confluence decreased, reaching a minimum when CMs were stored at 37°C (Figure 1A). Interestingly, the proliferation of eACs generally tended to increase with the MSC-CM storage duration (Figure 1B), but no statistical evidence supported this observation. Live imaging carried out during proliferation assays did not show any modification in eAC morphology, but some aggregates were observed in CMs stored at −20°C and in some 20°C replicates (Supplementary Figure S3). There is no standard deviation for the 20°C condition at 1 month of storage, because we could not obtain reliable counts for this condition in one of the experiments. Overall, the improved proliferation with MSC-CM was best after storage at −80 and −20°C.

**FIGURE 2:**
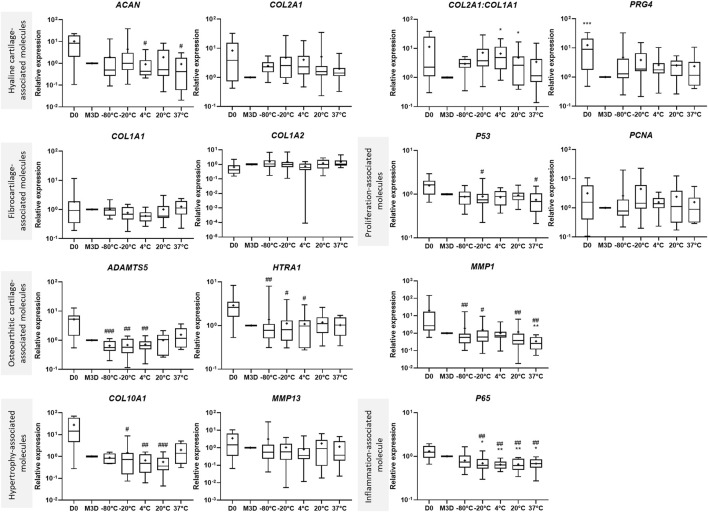
Storage temperature modulates the effects of CM on gene expression. CMs from BM-MSCs were harvested, filtered, aliquoted and stored at different temperatures. eACs (P2) were seeded in collagen sponges at 800,000 cells/sponge and then cultured in hypoxia. After 17 h, eACs were treated with CMs for 14 days. Then, sponges were harvested, washed twice with PBS and stored at −80°C. Total RNA was extracted and RT-qPCRs were carried out to assess gene expression. The expression of target genes was normalized using the reference genes *β-ACTIN* or *PPIA*. The D0 condition corresponds to eACs cultured in monolayer until P2. Experiments were reproduced with different strains of eACs and BM-MSCs (n = 12). Values are shown as box plots (median, quartiles, extreme values and mean (indicated with a "+") and were analyzed using the Kruskal-Wallis and the Dunn’s tests. *p < 0.05, **p < 0.01, ***p < 0.005, significantly different from the M3D condition. #*p* < 0.05, ##*p* < 0.01, ###*p* < 0.005, significantly different from the D0 condition. BM-MSCs: bone marrow-mesenchymal stromal cells; CM: conditioned medium; eACs: equine articular chondrocytes; M3D: control medium with 2% FBS; P2: passage 2; RT-qPCR: reverse transcription-quantitative polymerase chain reaction.

**FIGURE 3:**
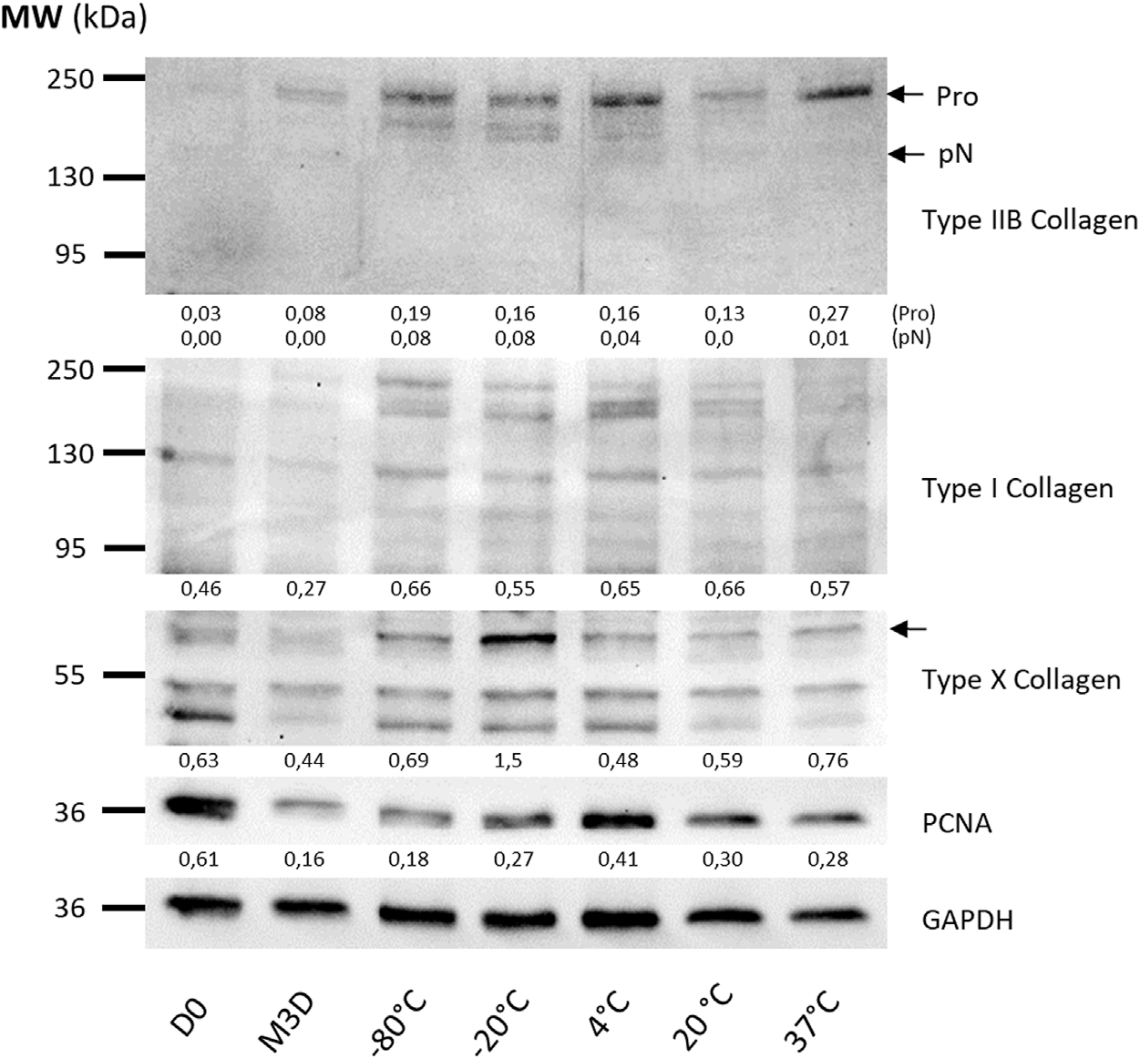
The effect of CM on ECM and Pcna protein levels depends on storage temperature. CMs from BM-MSCs (P3) were harvested, filtered, aliquoted and stored at different temperatures. eACs (P2) were seeded in collagen sponges at 800,000 cells/sponge and then cultured in hypoxia. After 17 h, eACs were treated with CMs for 14 days. Then, sponges were harvested, washed twice with PBS and stored at −80°C. The total proteins were extracted using a dedicated buffer supplemented with protease inhibitors, and western blots were used to evaluate protein levels, as shown in the representative images. The D0 condition corresponds to eACs cultured in monolayer until P2. Experiments were reproduced with different strains of eACs and BM-MSCs (n = 9). The densitometry of each band was measured thanks to the Image Lab software (Biorad). The relative values (relative to GAPDH) are indicated below each blot. BM-MSCs: bone marrow-mesenchymal stromal cells; CM: conditioned medium; eACs: equine articular chondrocytes; M3D: control medium with 2% FBS; P2: passage 2; P3: passage 3.

**FIGURE 4:**
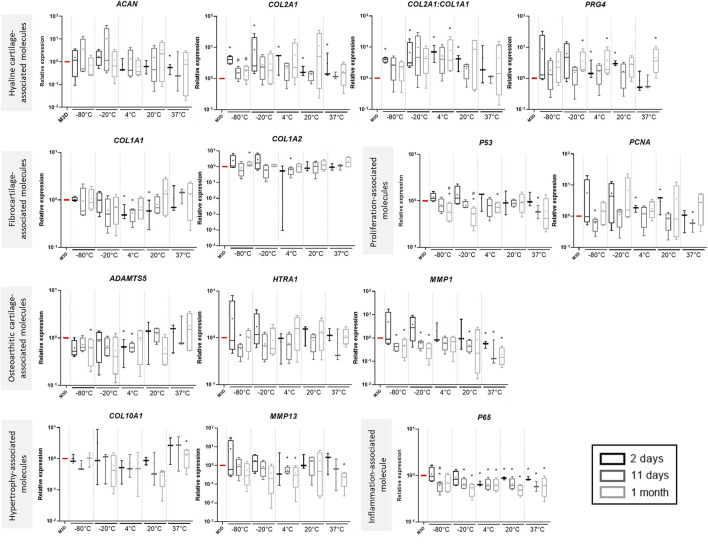
The effects of CM on gene expression can depend on storage duration. CMs from BM-MSCs were harvested, filtered, aliquoted and stored at different temperatures for 2 days, 11 days or 1 month. eACs (P2) were seeded in collagen sponges at 800,000 cells/sponge and then cultured in hypoxia. After 17 h, eACs were treated with CMs for 14 days. Then, sponges were harvested, washed twice with PBS and stored at −80°C. Total RNA was extracted and RT-qPCRs were carried out to assess gene expression. The expression of target genes was normalized using the reference genes *β-ACTIN* and *PPIA*. Experiments were repeated for each storage time with different strains of eACs and BM-MSCs (n = 4). Values are shown as box plots (median, quartiles, extreme values and mean (indicated with a “+”) and were analyzed using the Mann-Whitney test, *p < 0.05 significantly different from the M3D condition or the 2-day storage time for each temperature group (#*p* < 0.05). BM-MSCs: bone marrow-mesenchymal stromal cells; CM: conditioned medium; eACs: equine articular chondrocytes; M3D: control medium with 2% FBS; P2: passage 2; RT: room temperature; RT-qPCR: reverse transcription-quantitative polymerase chain reaction.

Subsequently, we evaluated the impact of storage temperature and time on the ability of the CMs to modulate the phenotype of the eACs (Figures 2, 3, 4). eACs were cultured as 3D cell cultures and differentially stored MSC-CMs were added to the cultures. The culture of eACs in the control M3D medium led to a decrease in hyaline cartilage, hypertrophy, osteoarthritic cartilage-associated molecules and *P65*, *P53* mRNA levels compared with D0 (untreated eACs at day 0) (Figure 2). After 14 days of culture, the MSC-CMs did not alter the *ACAN* mRNA levels of the eACs compared with eACs cultured in M3D (Figure 2). In contrast, *COL2A1* and *PRG4* levels tended to increase, for all storage temperatures. Moreover, the mRNA levels of *COL1A1 and COL1A2* tended to decrease when the CMs were stored at 4°C, but the levels of *COL1A1* tended to increase slightly when CMs were stored at 37°C (Figure 2). The functional *COL2A1:COL1A1* ratio was higher when eACs were cultured with MSC-CM stored at 20°C or below, compared with eACs cultured in the control medium (M3D). At the protein level, compared with the control condition (M3D), type IIB and type I collagens showed increased levels when eACs were cultured with CM (Figure 3). The pro form of the type IIB collagen corresponds to the immature form of the collagen containing both the C and N terminal propeptide, and the pN form corresponds to the collagen without the propeptide C-terminal. The eACs cultured with CM stored at 4°C or 37°C exhibit only the pro form of the type IIB collagen.

Regarding the protease mRNA levels, *HTRA1* remained unchanged compared with the control M3D condition or decreased compared with the D0 condition (Figure 2). For the catabolic enzymes, *MMP1* mRNA levels were unchanged when eACs were cultured with the CMs stored at 20°C or below, whereas the storage of the CM at 37°C led to a decrease in *MMP1*, compared with the eACs cultured with M3D (Figure 2). *ADAMTS5* mRNA amounts decreased only when the CM was stored at temperatures ranging from −80°C to 4°C, otherwise the ADAMTS5 levels remained unchanged. Regarding the hypertrophy-associated molecules, *COL10A1* and *MMP13* expression levels remained unchanged between eACs cultured in the presence of CMs or M3D. The type X collagen protein amount was increased regardless of the storage temperature, with a greater increase when CMs were stored at −20°C (Figure 3). Regarding the cell proliferation markers, *P53* mRNA levels decreased significantly when the eACs were cultured with CMs stored at −20°C or at higher temperatures. Pcna protein amounts and mRNA levels increased, especially when the CMs were stored from −20°C to 37°C (Figures 3, 4), suggesting that CMs increase eAC proliferation. In terms of p65 NFκB subunit expression, regardless of the storage temperature of the CMs, there was a downregulation of *P65* mRNAs*,* compared with the M3D condition. Altogether these data indicate that a storage temperature of the CM at 4°C or below best favors collagen protein accumulation, maturation and cell proliferation.

To study the impact of storage time on the properties of CMs, we examined the results that we obtained for each CM storage duration (Figure 4). When the CMs were stored at negative temperatures, the shortest time storage (2 days) resulted in the highest increase in the *COL2A1* and *PRG4* mRNA levels. On the other hand, the CMs stored at positive temperatures for 2 days tended to lead to decreased *COL1A1* mRNA levels. Nevertheless, the *COL2A1:COL1A1* ratio remained increased or similar compared to the M3D control, regardless of the storage time. For all temperature groups, the MSC-CMs had a similar effect on *ADAMTS5* and *HTRA1* mRNA levels for all MSC-CM storage times and remained unchanged compared with M3D. Conversely, when the MSC-CMs were stored at negative temperatures, a 2-day storage time led to an increase in *MMP1, MMP13* and *HTRA1* levels compared with eACs cultured in the control medium (M3D), although these transcripts remained unaffected or lowered when eACs were cultured in presence of MSC-CMs stored for 11 days or 1 month. *COL10A1* levels varied little with storage time. For the eACs cultured in MSC-CM stored at negative temperatures, *P65* and *P53* levels decreased only in the 11-day and 1-month storage times. eACs cultured with MSC-CMs stored at positive temperatures had lower *P65* mRNA levels for all storage durations. Moreover, the effects of the CMs on *PCNA* mRNA levels also depended on storage duration, with an increase observed after 2 days of storage at temperatures ranging from −80°C to 20°C.

### Priming with equine IL-1β, TNF-α or IFN-γ induces gene expression of growth factors and immunomodulatory markers in MSCs

**FIGURE 5:**
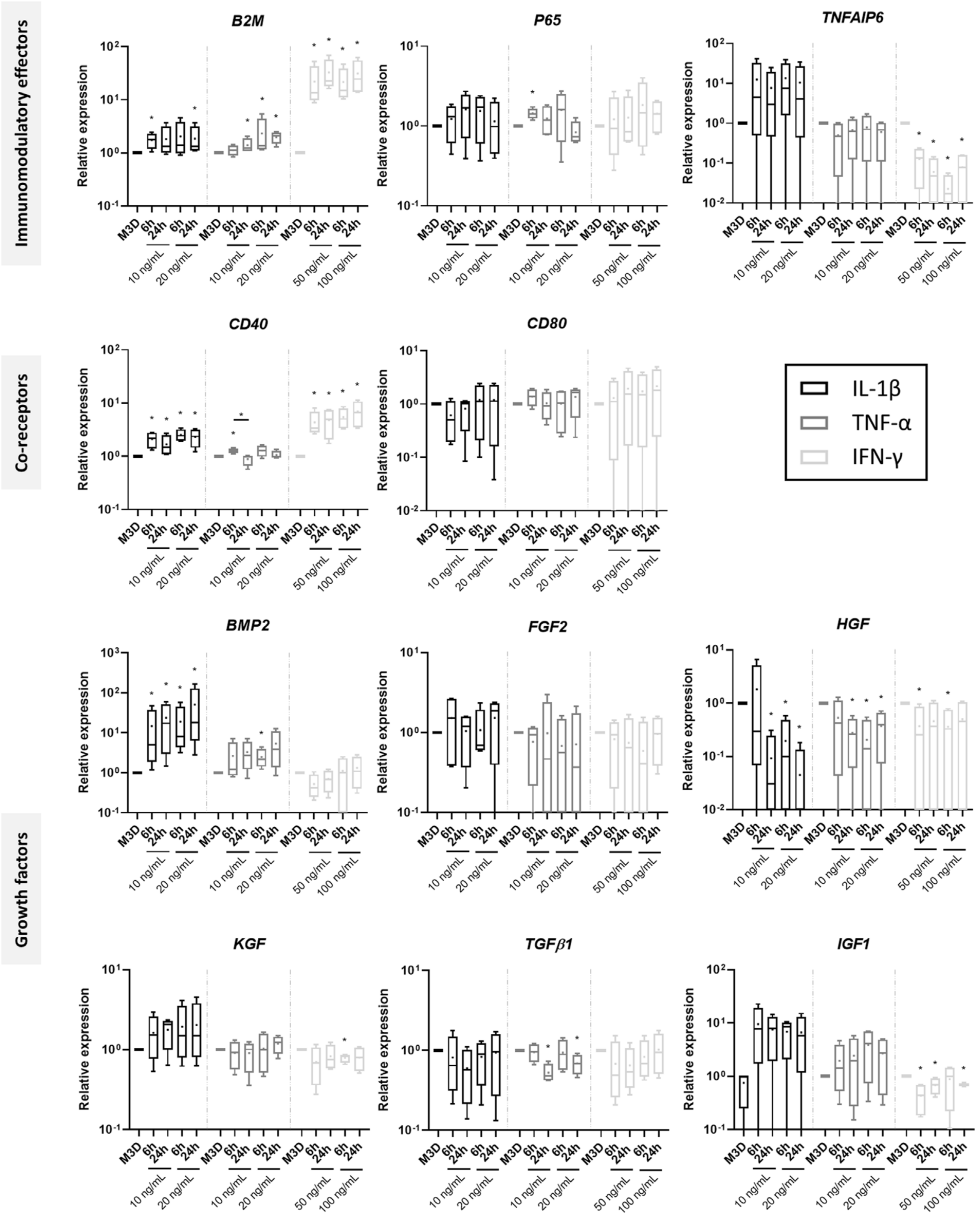
Effect of cytokine priming on the gene expression of immunomodulatory markers. At 50% confluency, MSC amplification medium with or without IL-1β (10 and 20 ng/mL), TNF-α (10 and 20 ng/mL) or IFN-γ (50 and 100 ng/mL) were added to BM-MSC cultures (P3) for 6 or 24 h (n = 4). After two washes with PBS, media were replaced with M3D for 24 h. Then, media were removed, MSCs were washed twice again with PBS and stored at −80°C until RNA extraction. The gene expression of several immunomodulatory markers was assessed using RT-qPCRs, and normalized using the reference genes *β-ACTIN* and *PPIA*. Values are shown as box plots (median, quartiles, extreme values and mean (indicated with a “+”) and were analyzed using the Mann-Whitney test, *p < 0.05, significantly different from the M3D condition within each cytokine priming group. BM-MSCs: bone marrow-mesenchymal stromal cells; M3D: unconditioned medium; CM: conditioned medium; eACs: equine articular chondrocytes; M3D: control medium with 2% FBS; P3: passage 3.

Based on a literature review, we evaluated two concentrations for each cytokine—10 and 20 ng/mL for IL-1β and TNF-α, 50 and 100 ng/mL for IFN-γ—and two priming times—6 and 24 h. MSC gene expression of the typical immunomodulation-associated molecules was assessed with RT-qPCRs (Figure 5). In comparison with the control condition (M3D), β2-microglobulin (*B2M*) mRNA levels of MSCs increased regardless of the priming time and the cytokine used, with a higher increase with IFN-γ. Moreover, IFN-γ significantly decreased TNFα-induced protein 6 (*TNFAIP6*) levels, whereas IL-1β and TNF-α did not significantly change its expression. *P65* increased significantly only for a priming duration of 6 h with 10 ng/mL of TNF-α. Regarding the co-receptors involved in lymphocyte activation, the levels of the cluster of differentiation 40 (*CD40*) also increased with IFN-γ or IL-1β priming. On the contrary, priming did not modulate the mRNA levels of CD80, regardless of the cytokine used. Variations in growth factor expressions were also observed. mRNA levels of *BMP2* and *IGF1* increased significantly or tended to increase with IL-1β or TFN-α priming. IFN-γ did not modulate the *BMP2* mRNA amounts and decreased the *IGF1* mRNA levels. *IDO1* mRNA levels remained undetectable except for MSCs cultured with IFNγ-primed CMs (Supplementary Figure S4). *INOS* transcript expression remained undetectable in MSCs cultured with the control medium, but was induced when MSCs were treated with primed CMs, although slight differences were observed between strains.

All cytokine priming methods significantly decreased the hepatocyte growth factor (*HGF*) levels, except the IL-1β priming at 10 ng/mL for 6 h. Transforming growth factor β1 (*TGFβ1*) levels tended to decrease with MSC priming; nevertheless, the difference was only significant for TNF-α priming for 24 h. *FGF2* and *KGF* remained unaffected by priming. *CD86*, *BMP6*, *IFN-γ*, *IL-1β*, *IL-10* and *TNF-α* immunomodulation-related gene expressions were also assessed, but the sensitivity of the method was insufficient to obtain any signal detection (data not shown).

Altogether, these results indicate that the three cytokines chosen for the MSC priming can modulate the gene expression of MSC immunomodulators, although each cytokine showed a different pattern MSC immunomodulation. Based on these results and for the next part of the study, we primed MSCs for 24 h with IL-1β at 20 ng/mL, TNF-α at 10 ng/mL and IFN-γ at 100 ng/mL.

### Pro-inflammatory cytokine priming affects the molecular composition of MSC-CM cytokines and chemokines

**FIGURE 6:**
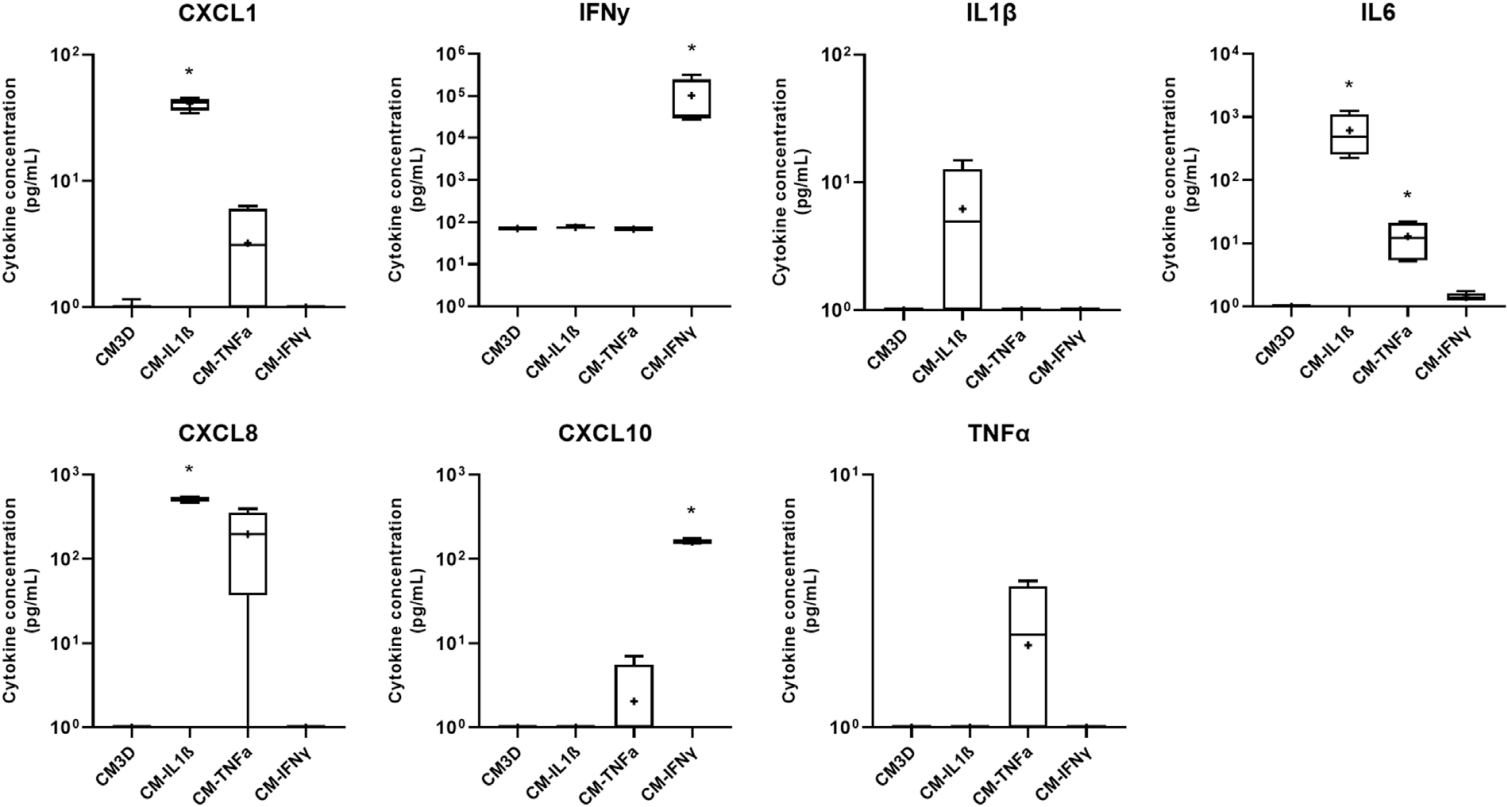
Priming of BM-MSCs with cytokines leads to an increase in pro-inflammatory molecule concentration in the CM. At 50% confluency, MSC amplification medium only or containing either IL-1β (20 ng/mL), TNF-α (10 ng/mL) or IFN-γ (100 ng/mL) was added to BM-MSC cultures (P3) for 24 h. Media were then removed, cells were washed twice with PBS and M3D was added. After 24 h, media were collected and stored at −80°C. Dosages of cytokines were performed using the MILLIPLEX^®^ Equine Cytokine/Chemokine Magnetic Bead assay (Merck-Millipore) according to the manufacturer’s recommendations. Measurements were performed in duplicate with the Luminex^®^ MAGPIX^®^ CCD imager and processed with the Luminex^®^ xPONENT software (n = 4). Values are shown as box plots (median, quartiles, extreme values and mean (indicated with a “+”) and were analyzed using the Mann-Whitney test, *p < 0.05 significantly different from the unprimed CM3D condition. eACs: equine articular chondrocytes; BM-MSCs: bone marrow-mesenchymal stromal cells; CM3D: (unprimed) conditioned medium.

We then primed MSCs with pro-inflammatory cytokines to enhance their immunomodulatory capacities and assessed whether this priming affects the concentration of pro-inflammatory molecules in the CMs. Of the 23 cytokines tested (Supplementary Table S1), only seven cytokines reached the detection thresholds (see Figure 6). Except for chemokine (C-X-C motif) ligand (CXCL) 1 and IFN-γ, none of the tested cytokines were detected in the CM3D condition, which corresponds to naïve MSC-derived CM. IL-1β priming significantly increased CXCL1, IL6 and CXCL8 concentrations and tended to slightly increase the IL1-β concentration in CMs. TNF-α priming also increased the concentration of IL6, and tended to increase the concentrations of CXCL8, CXCL10 and TNF-α in the CMs. IFN-γ priming led to a drastic 100- and 1000-fold higher concentrations of CXL10 and IFN-γ, respectively, in the primed CM compared with the unprimed control CM3D condition. Finally, we checked if priming with IL1-β increases the BMP2 concentration in the medium, as observed at the mRNA level (Figure 5). Unfortunately, the BMP2 levels remained too low to be detected with an enzyme-linked immunosorbent assay (ELISA) (data not shown).

Overall, the priming did not decrease the concentration of the pro-inflammatory cytokines studied, and even increased the concentration of the cytokines used for MSC priming.

### Priming does not significantly modulate CM pro-proliferative capacities

**FIGURE 7:**
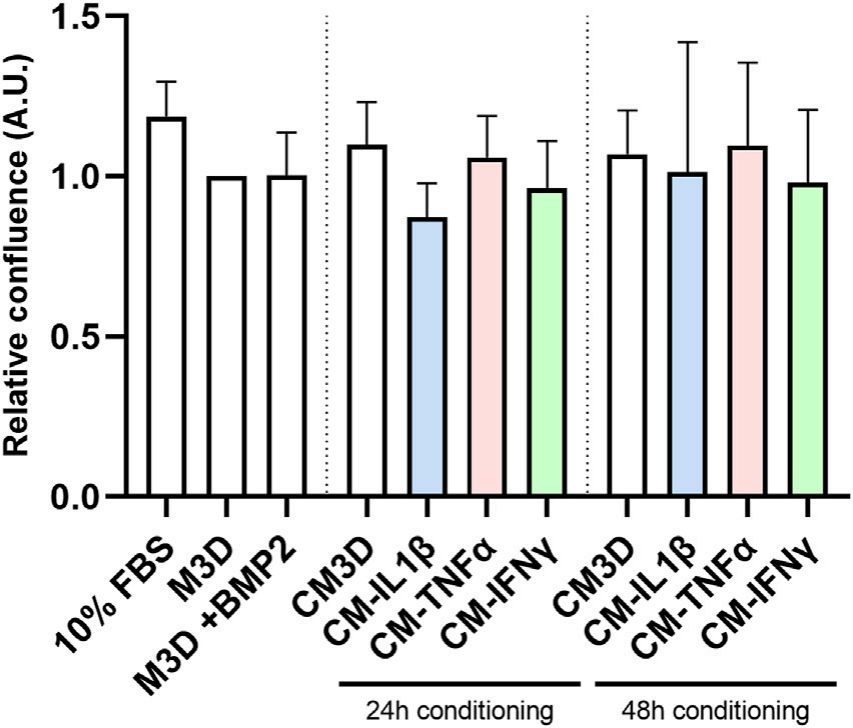
Priming with pro-inflammatory cytokines does not impact the eAC proliferation rate. BM-MSCs (P3) were incubated with IL-1β (20 ng/mL), TNF-α (10 ng/mL) or IFN-γ (100 ng/mL) for 24 h. Then, cells were washed twice with PBS and M3D was added to cultures for 24 or 48 h. CMs from BM-MSCs were harvested, filtered, aliquoted and stored at −80°C. In parallel, eACs (P2) were seeded in monolayer at 20,000 cells/cm^2^ in a 96-well plate. After 16 h, the medium was removed, cells were washed with PBS and treated with CMs. Cultures were monitored for 48 h using an Incucyte^®^ live imaging system. Each condition was tested in triplicate, and cells were counted on 3 representative areas of each image taken just after CM addition and after 48 h of treatment. Relative cell confluence was determined from the ratio of the (number of cells at 48 h to the number of cells at 0 h. Histograms show the impact of cytokine priming on the eAC proliferation rate over all experiments (n = 5). Mean values are shown as histograms ± standard deviation, and were analyzed using the Mann-Whitney test to compare the difference with the M3D condition. BM-MSCs: bone marrow-mesenchymal stromal cells; A.U.: arbitrary unit; BMP2: bone morphogenetic protein 2; CM: conditioned medium; eACs: equine articular chondrocytes; FBS: fetal bovine serum; M3D: control medium with 2% FBS; P2: passage 2; P3: passage 3.

eAC relative confluence was assessed based on cell counts after 48 h of culture in the presence of CMs (Figure 7). There were no statistically significant differences between any of the conditions. eACs grown in 10% FBS medium displayed a higher relative confluence than eACs cultured in all the other media, containing only 2% FBS. Unprimed CM (CM3D) slightly increased the proliferation of eACs, compared with eACs cultured with the control medium (M3D). The CM primed with IL-1β and conditioned for 24 h, with or without BMP2, tended to slightly decrease eAC relative confluence compared with the unprimed control (CM3D) condition (Figure 7, Supplementary Figure S5). Priming with TNF-α or IFN-γ did not modulate the ability of the CM, used without BMP2, to enhance eAC proliferation, whatever the conditioning time. On the contrary, the CMs obtained from MSCs primed with TNF-α or IFN-γ for 48 h and used with BMP2 tended to decrease the ability of the CM to enhance eAC proliferation, even though this decrease was not statistically significant.

### MSC preconditioning induces modulation of the expression of cartilage-related genes in eACs

**FIGURE 8:**
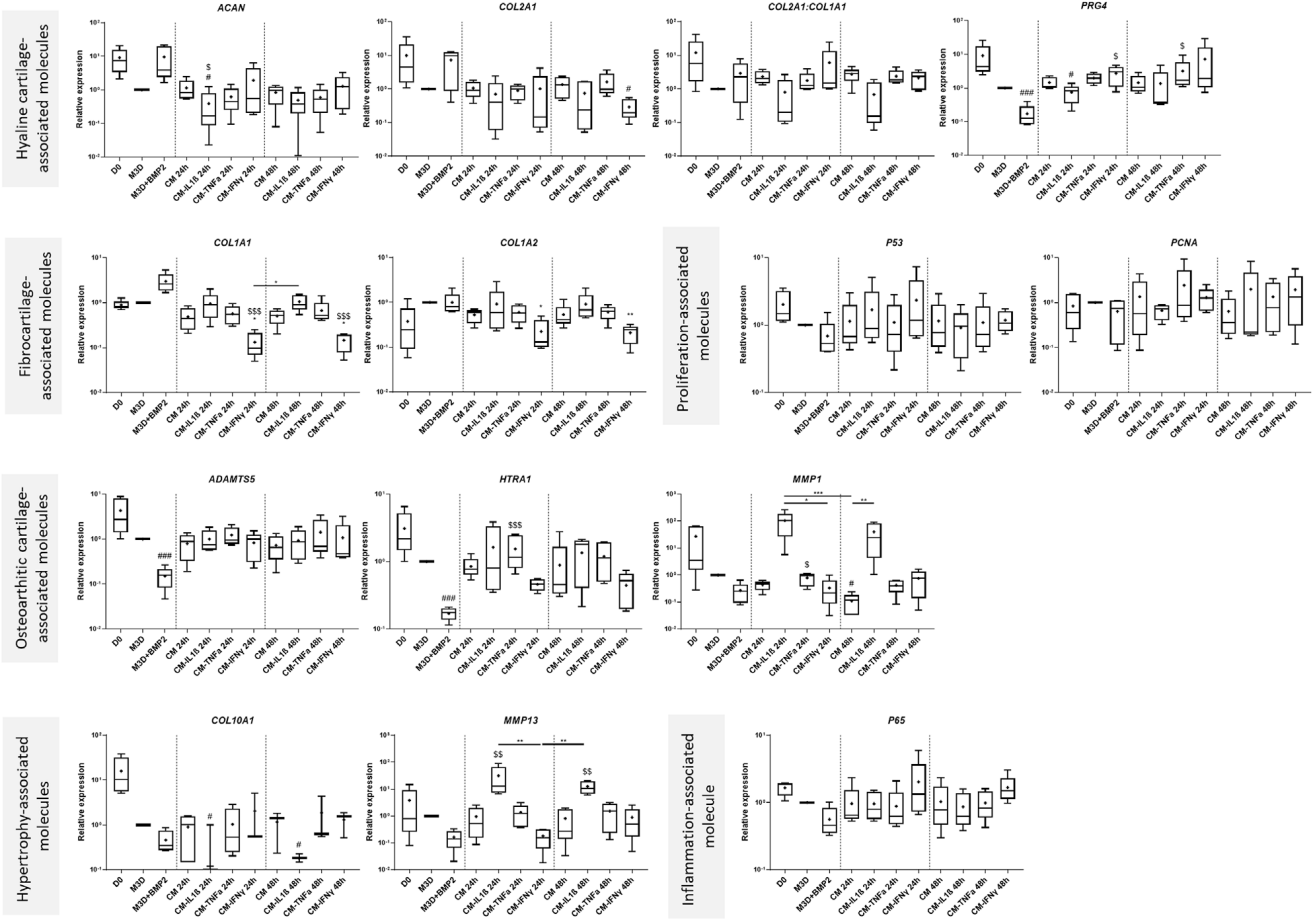
Primed CMs differentially modulate the gene expression of cartilage and OA-associated molecules in eACs. BM-MSCs (P3) were incubated with IL-1β (20 ng/mL), TNF-α (10 ng/mL) or IFN-γ (100 ng/mL) for 24 h. Then, cells were rinsed twice with PBS and M3D was added to cultures for 24 or 48 h. CMs from BM-MSCs were harvested, filtered, aliquoted and stored at −80°C. In parallel, eACs (P2) were seeded in collagen sponges at 800,000 cells/sponge and, after 17 h, were treated with CMs for 14 days. Then, sponges were harvested, washed twice with PBS and stored at −80°C. Total RNA was collected from these cultures and RT-qPCRs were carried out to assess gene expression. The expression of target genes was normalized using the reference genes *β-ACTIN* and *PPIA*. The D0 condition corresponds to eACs cultured in monolayer until P2. Experiments were repeated with different strains of eACs and BM-MSCs (n = 5). Values are represented as box plots (median, quartiles, extreme values and mean (indicated with a "+") and were analyzed using the Kruskal-Wallis and the Dunn’s tests. *p < 0.05, **p < 0.01, ***p < 0.005, significantly different from the M3D condition. #*p* < 0.05, ##*p* < 0.01, ###*p* < 0.005, significantly different from the D0 condition. $ *p* < 0.05, $$ *p* < 0.01, $$$ *p* < 0.005, significantly different from the M3D + B condition Note: BM-MSCs: bone marrow-mesenchymal stromal cells; BMP2: Bone morphogenetic protein 2; CM: conditioned medium; eACs: equine articular chondrocytes; M3D: control medium with 2% FBS; P2: passage 2; P3: passage 3; RT-qPCR: reverse transcription-quantitative polymerase chain reaction.

We have previously shown that MSC-CM can induce collagen synthesis (Contentin et al., 2022). We thus tested whether the CMs harvested from primed MSCs can enhance ECM production. To do so, eACs were cultured as 3D cell cultures with the CMs obtained from primed MSCs or naïve MSCs. eACs cultured with CMs conditioned for 24 h or 48 h from MSCs primed with IL1β (CM-IL1β) tended to have lower *ACAN* mRNA levels, unchanged *COL2A1* and *PRG4* levels, but higher *COL1A1 and COL1A2* levels than eACs cultured with unprimed CMs (Figure 8). With the CMs from MSCs primed with IL-1β and BMP2, the levels of *ACAN* and *COL2A1* were lower, whereas the levels of *COL1A1, COL1A2* and *PRG4* were similar between the unprimed CM and CM-IL1β conditions (Supplementary Figure S6). Therefore, with or without BMP2, the *COL2A:COL1A1* ratio decreased (Figure 8, Supplementary Figure S6). With or without BMP2, the CM-IL1β tended to induce the upregulation of *HTRA1*, *MMP1*, *MMP13*, whereas the levels of *ADAMTS5*, *P53* and *P65* remained unchanged compared with the unprimed condition. Interestingly, the levels of the hypertrophy-associated molecule *COL10A1* were reduced in the presence of CM-IL1β (Figure 8, Supplementary Figure S6). *PCNA* mRNA amounts decreased when eACs were cultured with the CM-IL1β conditioned for 24 h compared with eACs cultured with unprimed CM, which is correlated with the proliferation assay (Figures 2, 8).

eACs cultured with CM or CM-TNFα had similar levels of *ACAN*, *COL2A1,* but tended to have decreased *COL1A1* and *COL1A2* levels compared with eACs cultured in M3D (Figure 8, Supplementary Figure S6). In the presence of CM-TNFα, without BMP2, *PRG4* levels and the *COL2A1:COL1A1* ratio were higher than in the M3D condition. CM-TNFα did not have any drastic effects on the other transcripts studied. Regarding priming with IFNγ, CM-IFNγ did not modulate the levels of *PRG4*, *ACAN*, compared with eACs cultured in unprimed CM. For the CM condition without BMP2, the *COL2A1* levels remained unchanged when the CM-IFNγ was conditioned for 24 h but decreased for the 48-h conditioning. Additionally, in the presence or absence of BMP2, *COL1A1* and *COL1A2* levels were lower whatever the conditioning time. Thus, the *COL2A1:COL1A1* ratio tended to increase only when CM-IFNγ was conditioned for 24 h. With BMP2, CM-IFNγ did not modulate the mRNA levels of *PCNA*, *ADAMTS5*, *MMP1*, *HTRA1* compared with eACs grown in CMs, whereas the levels of the hypertrophy-associated molecules *P65* and *P53* increased (Supplementary Figure S6). Without BMP2, the same trends were observed, except for decreased *HTRA1* and *MMP13* levels (only after 24 h of conditioning for the latter) (Figure 8).

Altogether, these data underline that priming MSCs with inflammatory cytokines induces distinct effects on gene expression of cartilage-related molecules in eACs, according to the cytokine used.

### The eAC protein profile is enhanced by MSC secretome priming

**FIGURE 9:**
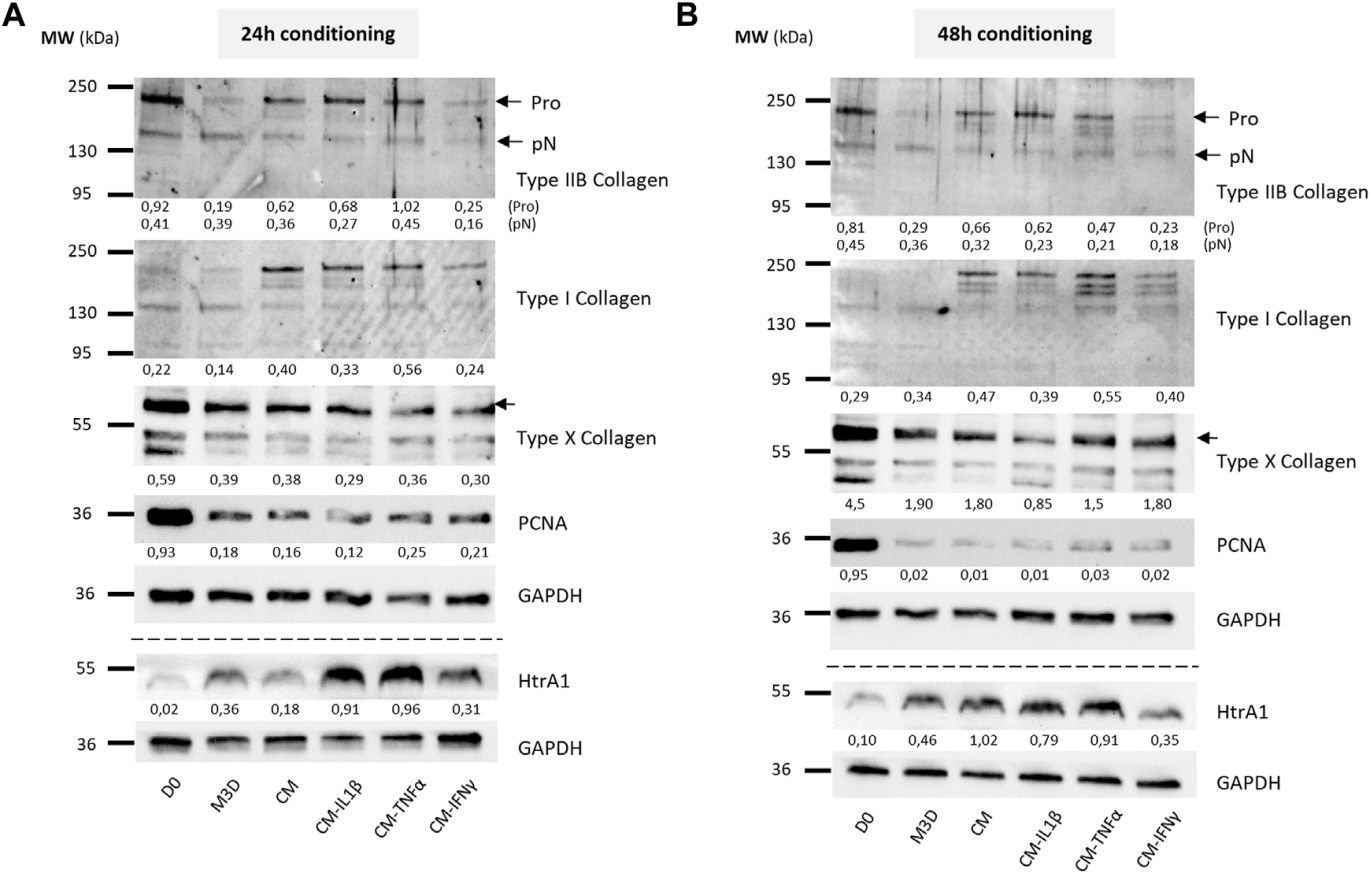
Priming of BM-MSCs with pro-inflammatory cytokines modulates protein accumulation of cartilage biomarkers in eAC cultures. BM-MSCs (P3) were incubated with IL-1β (20 ng/mL), TNF-α (10 ng/mL) or IFN-γ (100 ng/mL) for 24 h. Then, cells were rinsed twice with PBS and M3D was added to cultures for 24 **(A)** or 48 h **(B)**. CMs from BM-MSCs were harvested, filtered, aliquoted and stored at −80°C. In parallel, eACs (P2) were seeded in collagen sponges at 800,000 cells/sponge and, after 17 h, were treated with CMs for 14 days. Then, sponges were harvested, washed twice with PBS and stored at −80°C. Total proteins were extracted using a dedicated buffer supplemented with protease inhibitors and western blots were run to evaluate protein levels, as shown in the representative images. The D0 condition corresponds to eACs cultured in monolayer until P2. Experiments were repeated with different strains of eACs and BM-MSCs (n = 5). The densitometry of each band was measured thanks to the Image Lab software (Biorad). The relative values (relative to GAPDH) are indicated below each blot. BM-MSCs: bone marrow-mesenchymal stromal cells; CM: conditioned medium; eACs: equine articular chondrocytes; M3D: control medium with 2% FBS; P2: passage 2; P3: passage 3.

**FIGURE 10:**
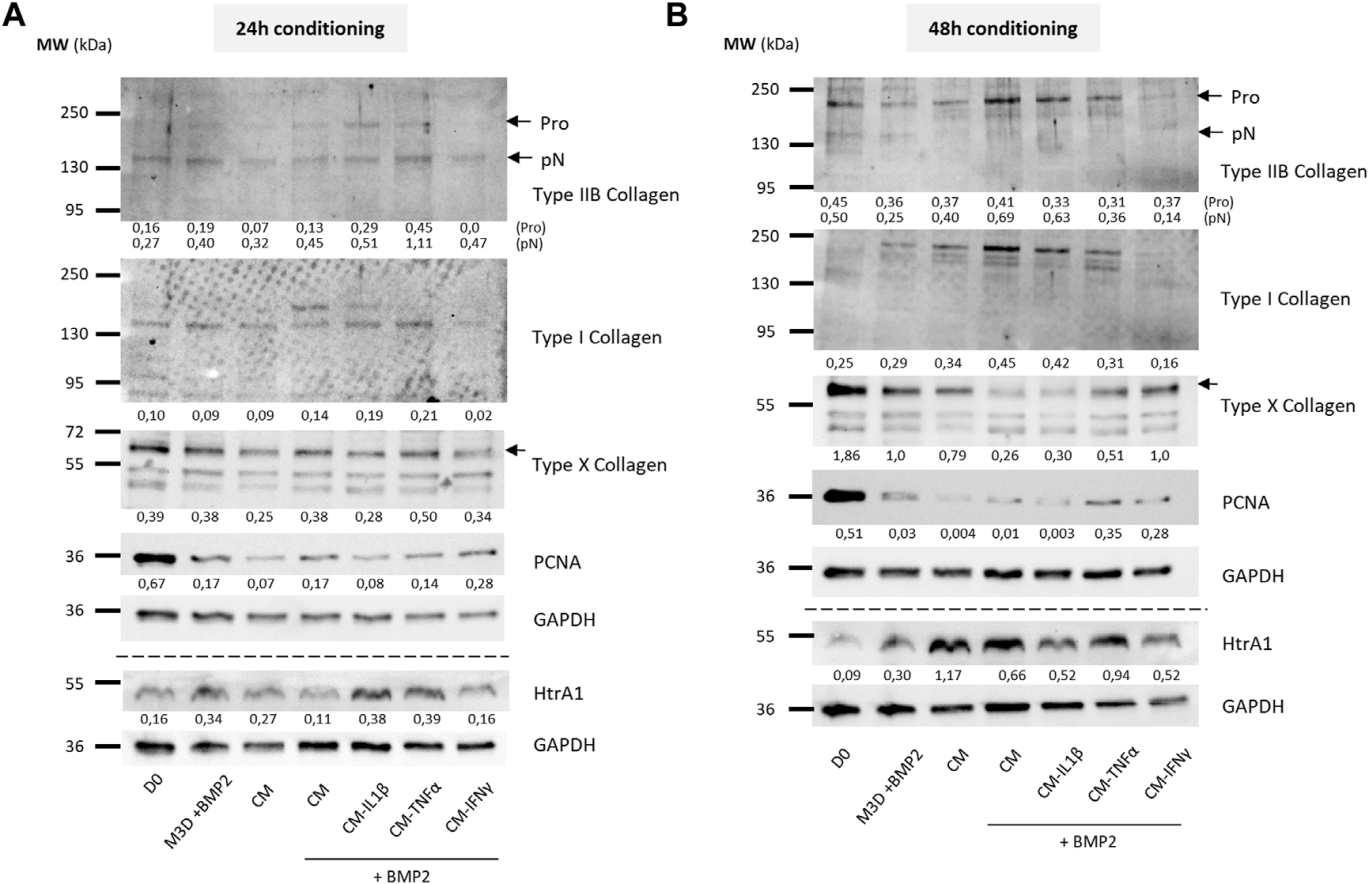
Priming of BM-MSCs with pro-inflammatory cytokines modulates protein accumulation of cartilage biomarkers in eAC cultures, in the presence of BMP2. BM-MSCs (P3) were incubated with IL-1β (20 ng/mL), TNF-α (10 ng/mL) or IFN-γ (100 ng/mL) for 24 h. Then, cells were rinsed twice with PBS and M3D was added to cultures for 24 **(A)** or 48 h **(B)**. CMs from BM-MSCs were harvested, filtered, aliquoted and stored at −80°C. In parallel, eACs (P2) were seeded in collagen sponges at 800,000 cells/sponge and, after 17 h, were treated with CMs with or without BMP2 (50 ng/mL) for 14 days. Then, sponges were harvested, washed twice with PBS and stored at −80°C. Total proteins were extracted using a dedicated buffer supplemented with protease inhibitors and western blots were run to evaluate protein levels, as shown in the representative images. The D0 condition corresponds to eACs cultured in monolayer until P2. Experiments were repeated with different strains of eACs and BM-MSCs (n = 3). The densitometry of each band was measured thanks to the Image Lab software (Biorad). The relative values (relative to GAPDH) are indicated below each blot. BM-MSCs: bone marrow-mesenchymal stromal cells; CM: conditioned medium; eACs: equine articular chondrocytes; M3D: control medium with 2% FBS; P2: passage 2; P3: passage 3.

To evaluate the effect on eACs of CM primed with cytokines on the protein expression of cartilage biomarkers, eACs were cultured in naïve CMs or CMs conditioned for 24 h (Figure 9A, 10A) or 48 h (Figures 9B, 10B) from primed MSCs. After 14 days of *in vitro* culture, eACs in M3D expressed lowered type IIB collagen levels compared with naïve CMs that favored the accumulation of types IIB and I collagen. eACs cultured with CM-IL1β better preserved type IIB collagen amounts and slightly modulated type I collagen accumulation for both conditioning times (Figures 9A,B). In the presence of BMP2, CM-IL1β had the same effect on type IIB and type I collagens, except when the CM was conditioned for 48 h where the type IIB levels were slightly lower than the CM condition (Figures 10A,B). Regarding the hypertrophy-associated markers, the protein amounts of type X collagen were slightly lower when eACs were grown in CM-IL1β without BMP2 regardless of the conditioning time (Figures 9A, B). With BMP2, the type X collagen protein levels slightly decreased only when eACs were grown in CM-IL1β conditioned for 24 h (Figures 10A, B). With or without BMP2, Htra1 was higher only in the presence of CM-IL1β conditioned for 24 h, compared with eACs cultured in the unprimed CM (Figures 9A, B, 10A, B). eACs cultured with CM-TNFα conditioned for 24h, with or without BMP2, had higher type IIB and I collagen amounts as the eACs cultured with the unprimed CM. On the contrary, the Htra1 protein amount increased in CM-TNFα that had been conditioned for 24 h and 48 h (Figures 9A, B). Additionally, the CM-TNFα conditioned for 48 h led to a decrease in the type IIB collagen, type I collagen and an increase in the type X collagen protein amounts, compared with the eACs cultured with unprimed CMs (Figure 10B). Surprisingly, in the presence or absence of BMP2, the CM-IFNγ systematically led to a decrease in type I and type IIB collagen protein amounts, compared with the eACs cultured with the unprimed CM (Figures 9A, B, 10A, B). The CM-IFNγ conditioned for 48 h led to the decrease in the Htra1 amount and the increase in the type X collagen only in presence of BMP2 for the latter (Figures 9B, 10B). With or without BMP2, Pcna amounts were lower than in D0 condition, and no variations were observed between the conditions (Figures 9A, B, 10A, B).

Altogether these data show, in a cartilaginous 3D culture model, that the CM derived from MSCs primed with IL-1β better favored collagen accumulation, as well as protease expression. On the contrary, the CMs derived from MSCs primed with IFNγ had negative effects on collagen accumulation, whereas the CMs derived from MSCs primed with TNFα had more subtle effects on the collagen amounts.

## Discussion

The debilitating consequences of OA inevitably lead to impaired animal wellbeing and cause substantial financial losses in the equine industry. Current therapies struggle against ineffective cartilage regeneration potential, and, to date, no curative treatment is available either in horses or in humans. Horses and humans share similarities in articular cartilage and OA etiology. Therefore, exploring innovative treatments for equine OA may also benefit humans, particularly in the context of the “One Health” approach. MSC-based therapies have already demonstrated promising evidence of their potential, validated and fostered by the work in our research laboratories (Branly et al., 2018; Bertoni et al., 2021). However, some concerns inherent to cell therapy tend to promote investigations that favor cell-free strategies. The MSC secretome is known to mediate part of the MSC therapeutic effect. Thus, the MSC secretome represents an encouraging and more reliable alternative to capitalize on the MSC therapeutic potential, and nonetheless overcome the limitations inherent to cell therapy (Muhammad et al., 2019; Jammes et al., 2023). Collecting the MSC secretome *in vitro* is a key step to preserving its properties, and many options are available according to the biological sample type and the purpose of the study (Jammes et al., 2023). However, one of the easiest and most effective methods is conditioning the media of cells cultured in a monolayer. This well-established technique is widely used to harvest the secretome and has contributed to the acquisition of a large amount of original data regarding the therapeutic potential of the MSC secretome in equine cartilage engineering (Toh et al., 2017).

A growing number of studies have demonstrated the capacity of the MSC secretome to decrease inflammation, favor chondroprotection, improve cartilage histologically or increase the clinical score in OA (De Luna et al., 2020). However, to our knowledge, hyaline cartilage regeneration by an unmodified adult MSC-derived secretome remains to be demonstrated. Exosomes isolated from MSCs derived from the H1 human embryonic stem cell line can alleviate OA *in vivo* and increase type II collagen, while decreasing ADAMTS5, in murine chondrocytes cultured in a monolayer (Wang et al., 2017). As another example, Liu et al. demonstrated, in a rabbit articular cartilage defect model, that tissue regeneration is mediated by an MSC exosome-supplemented biomaterial. However, the newly formed type I collagen prevented the neosynthesized cartilage from being classified as hyaline (Liu et al., 2017).

### Optimization of CM storage time and temperature

Along with secretome collection, CM storage time constitutes another major aspect of storage protocols. Storage inevitably affects secretome components and many studies report aggregation and loss of biological material, especially for purified EVs (Jeyaram and Jay, 2017). Freshly collected CMs is logically the best method for using MSC-CMs, and our results were going this way, where the shorter storage time seems to promote hyaline cartilage and proliferation-associated molecules. However, short storage time is difficult to set up and need experimental and clinical requirements. Interestingly, we found that a storage temperature of the CM at 4°C or below downregulates inflammation, promotes cell proliferation, and collagen protein accumulation. Recent studies agree that EVs derived from MSCs are more likely to keep their original properties after storage at −80°C, although lyophilization and cryopreservation are increasingly considered alternatives (Qin et al., 2020; Yuan et al., 2021). Recently, the secretome stability of different cell types was confirmed after 6 months of storage at temperatures of up to 5°C (Laggner et al., 2020). Apart from this investigation, we did not find any other study testing the impact of storage temperature and time on whole secretome competence. In response to the lack of standardized guidelines on how to store unprocessed CMs, the first goal of this study was to determine the impact of storage temperature and time on equine MSC secretome capacities.

First, our results show that the profile of mRNA levels changed between the eAC seeding and 14 days in 3D cultures (D0 vs. M3D) which reflected the dedifferentiation process inherent to *in vitro* eAC culture. eAC dedifferentiation has previously been demonstrated in monolayer as well as in 3D *in vitro* amplification and is mainly characterized by the decrease in typical hyaline cartilage-associated marker levels (Lin et al., 2008; Ling et al., 2021). Here, we found that the MSC-CM stored at the lowest temperatures were the most effective in increasing eAC proliferation and ECM accumulation. On the contrary, the *PCNA* decrease in eACs cultured with CMs stored at the highest temperatures corroborated proliferation assay results. Nevertheless, −20°C storage increased protein accumulation of type X collagen, suggesting the hypertrophy of the eACs. CMs stored at this temperature also tended to be more prone to protein and/or EV aggregation, a well-known consequence of negative temperature conservation (Qin et al., 2020). This phenomenon can directly affect the distribution of secretome components in cell cultures, and thus influence their biological effect. Protein and EV aggregation can be minimized by adding stabilizers or cryoprotectants before storage, such as trehalose (Jeyaram and Jay, 2017). Further studies are needed to assess the impact of these stabilizers on the eAC phenotype.

Despite the lack of clear evidence in the literature, most protocols commonly assume that −80°C is the most appropriate temperature at which to preserve MSC-CM properties. Furthermore, low temperatures have been shown to promote the preservation of molecular activity and prevent compounds from degradation (Qin et al., 2020). Here, we confirmed that storage at −80°C is the best temperature for preserving the ability of CM to enhance eAC proliferation and ECM synthesis (type IIB and I collagen accumulation, increase in *PRG4* mRNA amounts and in the *COL2A1*:*COL1A1* ratio) and to downregulate inflammation. CM storage times of up to 1 month had minor effects on the ability of the CM to modulate phenotypic molecules/protease mRNA levels. However, longer storage times need to be tested, because they may be especially useful for clinical use.

### Optimization of cytokine priming

Therapeutic properties attributed to the MSC secretome are strongly associated with MSC immunomodulation abilities, which can be enhanced by multiple methods including cytokine priming (Shi et al., 2012). Additionally, a recent study demonstrated that cytokine priming helps attenuate the heterogeneity of MSC immunomodulatory capacities and could allow researchers to overcome this challenge (Szabó et al., 2015). IL-1β, TNF-α and IFN-γ are pleiotropic pro-inflammatory factors released by immune cells and actively take part in OA pathogenesis (Hunter and Bierma-Zeinstra, 2019; López-García and Castro-Manrreza, 2021). Despite their functional similarities, IFN-γ canonical signalization involves JAK/STAT pathway activation (Bhat et al., 2018), but TNF-α and IL-1β mainly exert their effects *via* the modulation of the nuclear factor-κB (NF-κB) pathway (Jenei-Lanzl et al., 2019; Webster and Vucic, 2020). MSCs are known to express the respective cytokine receptors and, therefore, should be able to trigger a cellular response in the presence of these cytokines (Li et al., 2016). IL-1β, TNF-α and IFN-γ are all found in OA synovial liquid and are strongly associated with OA-related inflammation (Hunter and Bierma-Zeinstra, 2019). Recent research has demonstrated the potency of these cytokines to trigger MSC activation (Colombini et al., 2019), even in the equine model (Barrachina et al., 2016; Barrachina et al., 2017a; Hill et al., 2017; Cassano et al., 2018; Caffi et al., 2020; Voskamp et al., 2020; Cequier et al., 2022). They can be used in synergy with lymphocyte stimulation to enhance MSC immunomodulation properties (Cequier et al., 2022). However, the MSC priming method must be carefully adjusted to maximize their therapeutic potential. Here, cytokines chosen for MSC priming were of equine origin to suit *in vivo* conditions and optimize the BM-MSC metabolic response. Indeed, human and equine cytokines do not share the same structural conformation, and, even if human molecules are generally more available and easier to purchase, using molecules from a human source on equine MSCs may lead to an inaccurate metabolic response. We found that MSC incubation times with each cytokine (6 h or 24 h) were not a critical parameter in the induction of immunomodulation-associated gene expression. Nevertheless, because variation in cytokine-induced gene expression was slightly more pronounced after 24 h of priming, we chose this priming duration to further investigate cytokine priming. Here, we demonstrated that all three priming molecules modulated immunomodulation-related gene expression; however, effects varied with the cytokine used. Other studies have demonstrated that TNF-α priming of MSCs induces an increase in FGF-2, IGF-1, and HGF concentration in CMs (Crisostomo et al., 2008); however, except for IGF-1, our results did not confirm this enhancement at the mRNA level. Regarding *HGF*, our results were more similar to those in the Caffi et al. study, which showed decreased expression of HGF in MSCs after 24 h of TNF-α or IFN-γ preconditioning (Caffi et al., 2020). TGF-β modulation also corresponded to those observed in our experiments. These discrepancies in the literature highlight the heterogeneity of the MSC metabolic response to cytokine priming, which may be due to MSC tissue origins or MSC population heterogeneity within a given niche (Jammes et al., 2023).

Numerous studies have primarily focused on the cytokine priming-induced anti-inflammatory MSC phenotype by demonstrating decreased pro-inflammatory mediator levels, overexpression of anti-inflammatory molecules or MSC-induced M2 macrophage polarization (Kudlik et al., 2016; Fernandes et al., 2020). However, changes in MSC immunoregulatory potential involve additional characteristics. For example, CD40 is implicated in T-cell activation and survival; nevertheless, its gene expression was significantly increased by priming with each of the three cytokines. The same patterns have been reported for *B2M* expression, an immunogenic molecule part of the major histocompatibility complex I (MHC-I). Here, we tested only the common immunomodulation-associated markers, some of which were not detected by RT-qPCR, which can easily be attributed to their low gene expression. Additionally, some of the assessed immune markers, such as TGF-β1 or IL-6, are known to have pleiotropic functions and cannot precisely be attributed to either a pro- or anti-inflammatory MSC response. Indeed, the final MSC response is presumably the result of a fine-tuned equilibrium of a wide range of molecules.

Here, cytokine dosages in CMs were assessed to determine if immune-related protein production reflected modulation of gene expression. The presence of pro-inflammatory molecules in CMs demonstrated MSC activation, but may also affect their preclinical or clinical use because these factors are implicated in OA pathogenesis. Furthermore, IL-1β, TNF-α and IFN-γ detection in CMs after priming, despite PBS washes, can originate from several events. First, immunomodulation is a complex process for which all signaling pathways have not been elucidated yet, and it remains possible that each of these three cytokines could have triggered their own auto-induction. Several studies have already demonstrated that pro-inflammatory priming increases gene expression of the same mediators, bringing us to the conclusion that auto-induction still needs to be considered (Barrachina et al., 2017b). Nevertheless, we were unable to detect IL-1β, TNF-α and IFN-γ at the mRNA level. Secondly, this phenomenon can also be explained by the cellular internalization of cytokines used for MSC priming, followed by their secretion into CMs during the medium conditioning step. Further studies are needed to investigate putative cytokine internalization/release trafficking. Indeed, most studies only focus on the MSC transcriptome or the release of soluble/EV-encapsulated pro-inflammatory mediators by MSCs without addressing the intake and trafficking of cytokines used for MSC priming (Barrachina et al., 2017b; Mardpour et al., 2019). Finally, the presence of these cytokines may be the result of molecular retention on the MSC surface that PBS washes did not succeed in eliminating. This hypothesis was also mentioned in a recent study that found higher levels of IL-1β in CMs primed with this cytokine (Even et al., 2022). These three hypotheses are not mutually exclusive and may explain our results. However, further investigations of cytokine dynamics are needed to fully understand the repercussions of priming on MSC secretome composition.

Our findings do not corroborate the transition of naïve MSCs toward either a pro- or anti-inflammatory phenotype. Nevertheless, all three priming cytokines modulated immunomodulation-associated molecules, confirming the activation of IL-1β, TNF-α and IFN-γ signaling as already demonstrated in equines (Barrachina et al., 2016; Caffi et al., 2020). Therefore, we used these cytokines to assess the capacity of primed CMs to enhance ECM synthesis/quality in cartilaginous 3D cultures.

### BM-MSC secretome priming improves the eAC phenotype

MSCs participate in numerous healing processes. Usually, MSCs are recruited and activated by inflammation-related factors, which makes cytokine priming an appropriate strategy for improving MSC properties. Most studies focus on MSC gene expression or secretome analysis to assess MSC immunomodulatory potential. However, the final therapeutic outcomes do not depend only on MSC immunomodulation properties and likely involve more complex mechanisms. The eAC phenotype and ECM composition are both critical factors to consider when confirming the beneficial effects of the MSC secretome with respect to cartilage engineering. Here, we demonstrated a slight positive effect of naïve CMs on the eAC proliferation rate, as previously (Contentin et al., 2022). Nevertheless, cytokine priming did not improve the capacity of the CMs to further increase eAC proliferation. Conversely, a weak negative impact of IL-1β preconditioning over naïve CM was observed, corroborated by RT-qPCR results that showed lower *PCNA* levels than in other conditions, but only for media conditioned for 24 h.

Regarding gene expression, only CMs derived from MSCs primed with TNF-α or IFN-γ led to an increase in the expression of cartilage ECM-associated molecules, with a stabilization or decrease of catabolism-related markers. We demonstrated that IL-1β priming decreased the functional *COL2A1:COL1A1* ratio through a decrease in *COL2A1*. Additionally, media conditioned from MSCs primed with IL-1β triggered a significant increase in *MMP1* and *MMP13* mRNA levels and HtrA1 protein amounts, which could be attributed to the increase in the concentration of pro-inflammatory molecules observed in the CM. IL-1β is one of the major pro-inflammatory cytokines involved in OA pathogenesis and its potential to increase *MMP1* and *MMP13* levels in eAC cultures has already been demonstrated (Bourdon et al., 2021; Cullier et al., 2022). However, the same studies have shown that the addition of IL-1β to eACs tended to be associated with decreased levels of *HTRA1*, which was not observed in our experiments. Nevertheless, protease activity is regulated by posttranslational regulation. We showed that eAC cultured in 3D with CM derived from MSCs primed with IL-1β—but not TNF-α or IFN-γ—exhibited higher type IIB collagen protein amounts. Simultaneously, we demonstrated that IL-1β priming led to a sharper increase in *BMP2* and *IGF1* gene expression in MSCs, both genes being involved in type II collagen expression (Zhang et al., 2009; Rakic et al., 2017). Thus, type II collagen induction by CM-IL1β may originate from the production of growth factors, such as BMP2, by MSCs. To verify our hypothesis, we performed an ELISA, but the, BMP2 levels were below the detection threshold. In any case, CM-IL1β may be of interest to help resume or maintain anabolism in OA.

Among the three cytokines, TNF-α priming showed the most discreet effects in our study. Despite its involvement in the development of OA, TNF-α has shown potential for use in OA management through its pro-chondrogenic capacities, which have been proven in MSC cultures (Voskamp et al., 2020). Here, we showed that CM-TNFα modulated eAC gene expression similarly to the naïve CMs. However, the decrease in the accumulation of some proteins, such as type I and IIB collagens and HtrA1, suggest a pro-catabolic effect of CM-TNFα on eAC metabolism.

Finally, CM-IFNγ induced a downregulation of collagen protein expression, particularly type IIB collagen, which could be partially explained by the decrease in *IGF1* levels in MSCs. In fact, IGFI is known to be highly associated with type IIB collagen regulation *via* the phosphoinositide 3-kinase (PI3K) pathway (Zhang et al., 2009). Additionally, CM-IFNγ led to the decrease of HtrA1 at the mRNA and protein levels. HtrA1 is known to inhibit signals mediated by TGF-β family members (Polur et al., 2010) carried by platelet-rich plasma (PRP)-based therapies (O’Connell et al., 2019; Del Amo et al., 2022), and substantially decreasing their therapeutic potential. Thus, combining PRP intra-articular injections with CM-IFNγ may potentiate the effect of PRP. Moreover, the decrease in *MMP1* and *MMP13* compared with naïve CM corroborates the promising potential of CM-IFNγ to help reduce the catabolism inherent to OA. Moreover, numerous studies have attempted to better mimic the *in vivo* environment and focus on cytokine combinations to optimize MSC immunomodulatory properties, currently mainly represented by the association of IFN-γ with TNF-α (Barrachina et al., 2017a; Hackel et al., 2021; López-García and Castro-Manrreza, 2021). Furthermore, combining CMs or EVs from different preparations and with complementary effects may enhance their respective capacities. For example, exosomes from MSCs primed with kartogenin recently proved their efficacy in countering type I collagen expression in chondrocytes (Liu et al., 2020). Associating these EVs with MSC-CM may help increase the type IIB to type I collagen ratio at the protein level, and restore a hyaline matrix composition in OA. Finally, it would be interesting to also combine the three cytokines IL-1, TNF- α, and IFNγ to potentialize the priming, in an attempt of synergic effect on MSC immunomodulatory properties. Also, additional analysis could be done to look at the quality of the extracellular matrix of the neosynthesized cartilage. For example, the level of production of proteoglycans could be evaluated by using Safranin-O staining or aggrecan immunostaining on the 3D culture.

Our previous work demonstrated that the MSC secretome favors collagen accumulation in a cartilaginous 3D culture model (Contentin et al., 2022). Herein, we hypothesized that the intensity of the therapeutic effect of the secretome on eACs is proportional to the amount of CM components, which, in turn, is probably dependent on conditioning time. However, we did not reveal drastic differences in gene expression between eACs cultured with CM conditioned 24 h or 48 h. One explanation is that the dose of the secreted molecules is already sufficient after 24 h of conditioning to trigger the optimum effects in eACs. However, this paradox can also be explained by a depletion in nutrients essential for eAC growth in the medium during the conditioning step, because MSCs used for secretome production continue to consume medium components. Thus, nutrient availability for the eACs decreases with the duration of the MSC conditioning time. However, long conditioning times are essential for attaining a satisfactory level of enrichment in the medium. Therefore, striking a balance between the quantity of nutrients and that of bioactive factors in the CM is the next challenge to address for CM use in therapeutics.

## Conclusion

This study provides essential practical information on the preservation of CM therapeutic potential and underlines the necessity of considering all experimental parameters to establish reliable, standardized protocols. Furthermore, our findings highlight the potential of equine IL-1β, TNF-α, and IFN-γ for MSC activation. Primed MSCs were able to generate bioactive factors of therapeutic interest to counter OA progression either by favoring anabolism or by attenuating catabolism. However, uncontrolled CM composition limits *in vivo* application possibilities and needs to be mastered for this innovative approach to reach its full potential. Many studies have shown the crucial involvement of EVs, and more precisely exosomes, in MSC secretome-mediated therapeutic potential (Toh et al., 2017; Ren et al., 2022). Thus, EVs stand out as a promising alternative to secretome use, because they mediate a large part of the MSC secretome. For instance, they could be used as a delivery system to carry bioactive factors and potentiate their therapeutic capabilities, limiting their degradation or elimination, and enhancing their positive impact on cartilage homeostasis. Further investigations are now needed to reach a high-scale production of MSC-derived biological material (Colombini et al., 2019).

To a greater extent, the MSC secretome may help enhance ACT (Brittberg et al., 1994). ACT requires *in vitro* chondrocyte amplification, which inevitably leads to chondrocyte dedifferentiation; therefore, this technique needs to be improved to reach its full potential. Preserving or even enhancing the chondrocyte phenotype by amplifying chondrocytes with bioactive factors that have a positive impact on their phenotype may offer a sustainable solution to avoid or at least limit chondrocyte dedifferentiation.

Finally, our findings supplement the newly accumulated knowledge on the therapeutic potential of the MSC secretome and actively contribute to the innovation effort to improve OA treatment. The use of the MSC secretome holds promise as a new therapeutic approach for equine OA management, thus beneficial for horse welfare and the equine industry in general. In addition, the horse is a good model for evaluating OA physiopathology or therapeutics, and according to the One Health approach, could also guide this original treatment method toward clinical applications in humans.

## Data Availability

The original contributions presented in the study are included in the article/Supplementary Material, further inquiries can be directed to the corresponding author.
