# Supplementary figures and images for: Effect of pro-inflammatory cytokine priming and storage temperature of the mesenchymal stromal cell (MSC) secretome on equine articular chondrocytes
Source: Front Bioeng Biotechnol. 2023 Aug 31;11:1204737. doi: 10.3389/fbioe.2023.1204737 (PMC10502223; doi:10.3389/fbioe.2023.1204737)

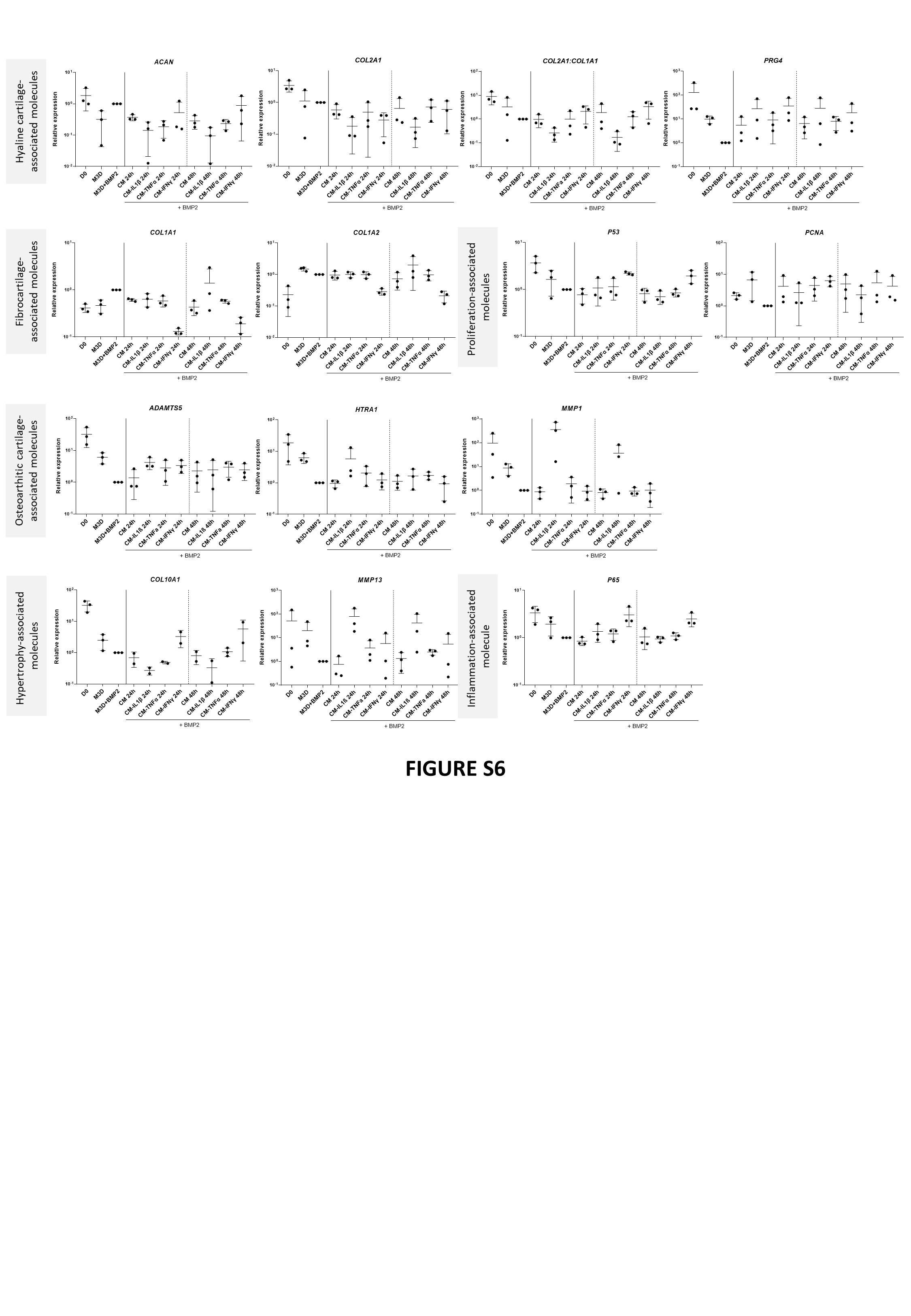

Supplement: Supplementary file 1 [file Image6.TIF]

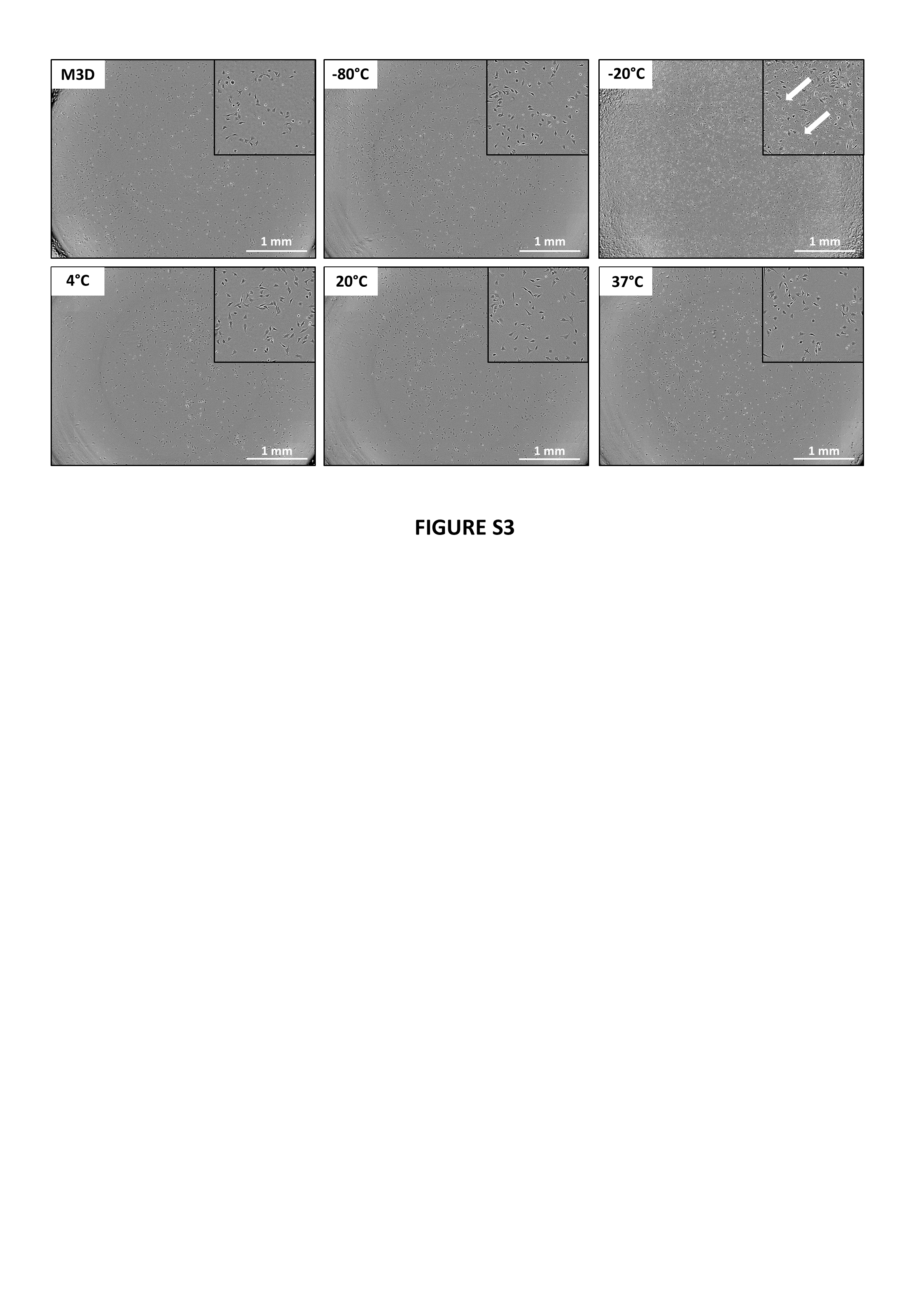

Supplement: Supplementary file 2 [file Image3.TIF]

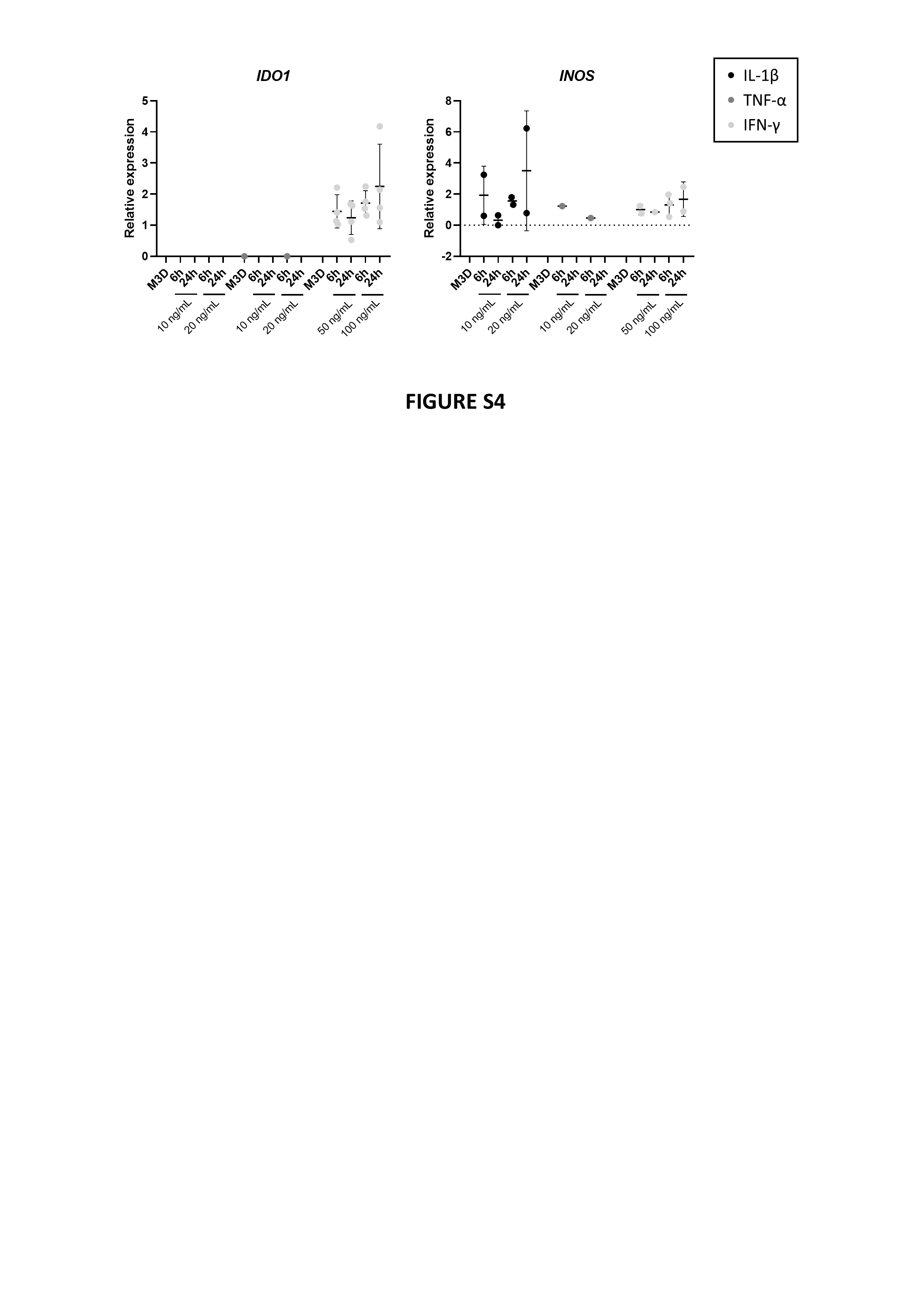

Supplement: Supplementary file 3 [file Image4.TIF]

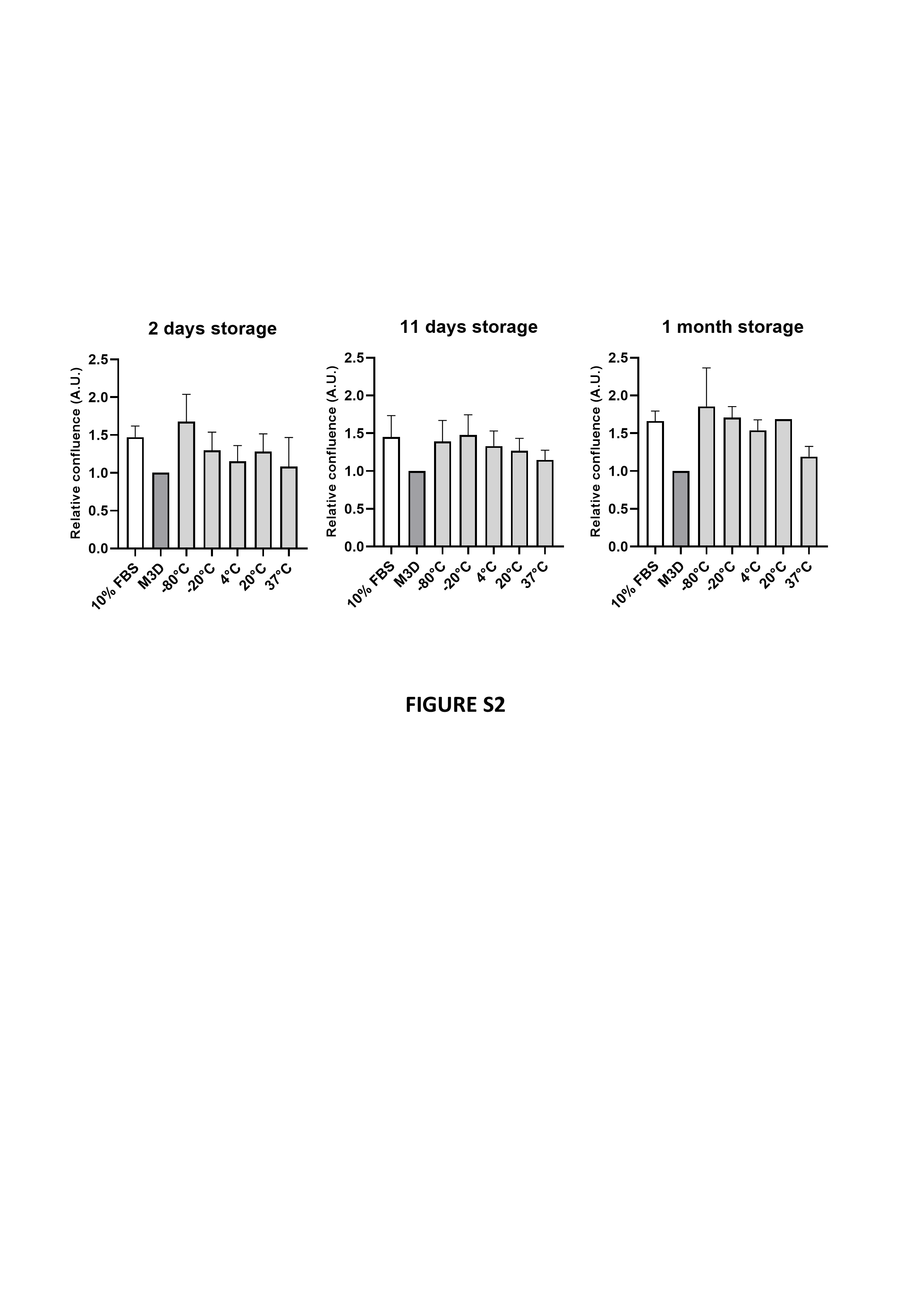

Supplement: Supplementary file 4 [file Image2.TIF]

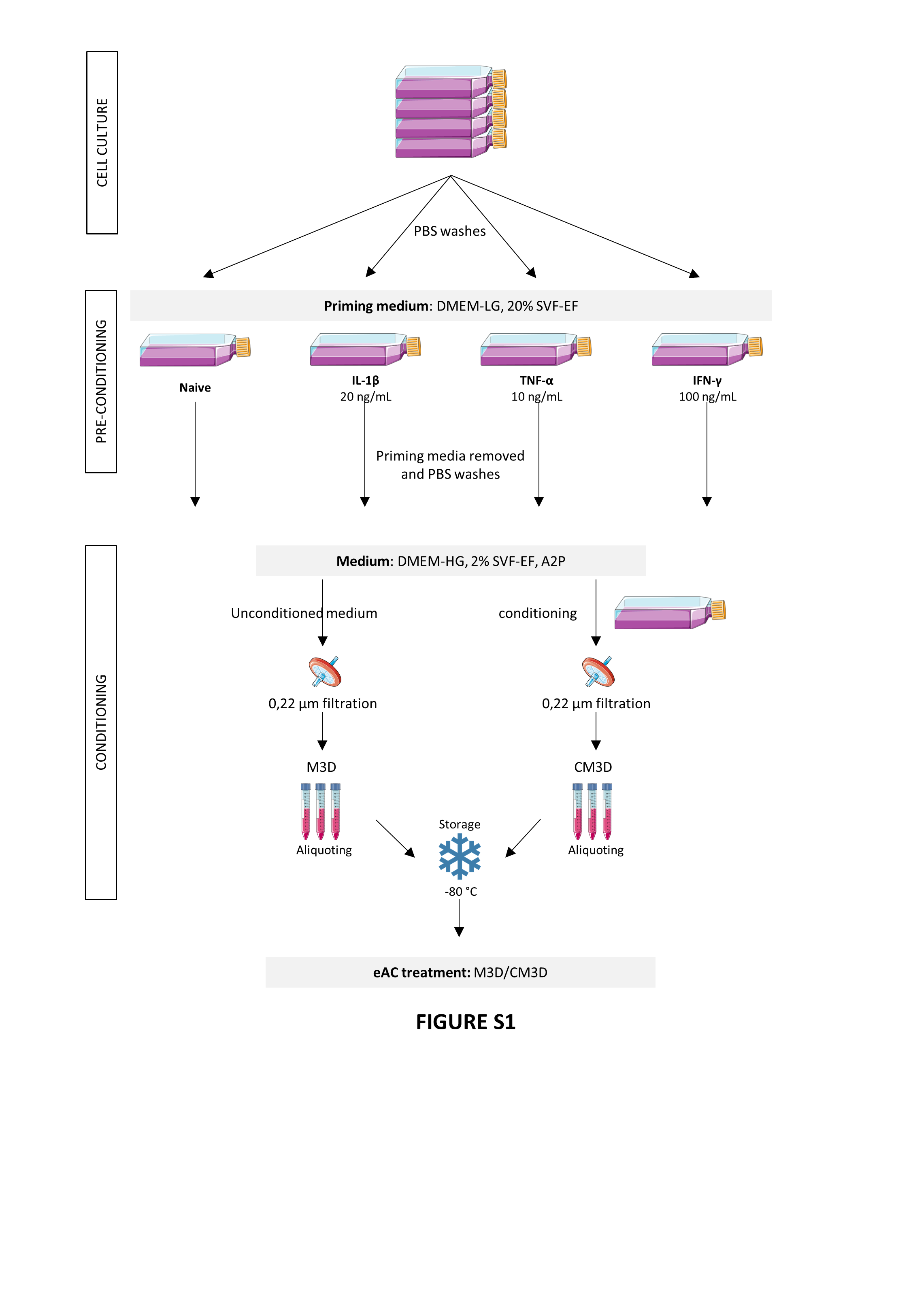

Supplement: Supplementary file 5 [file Image1.TIF]

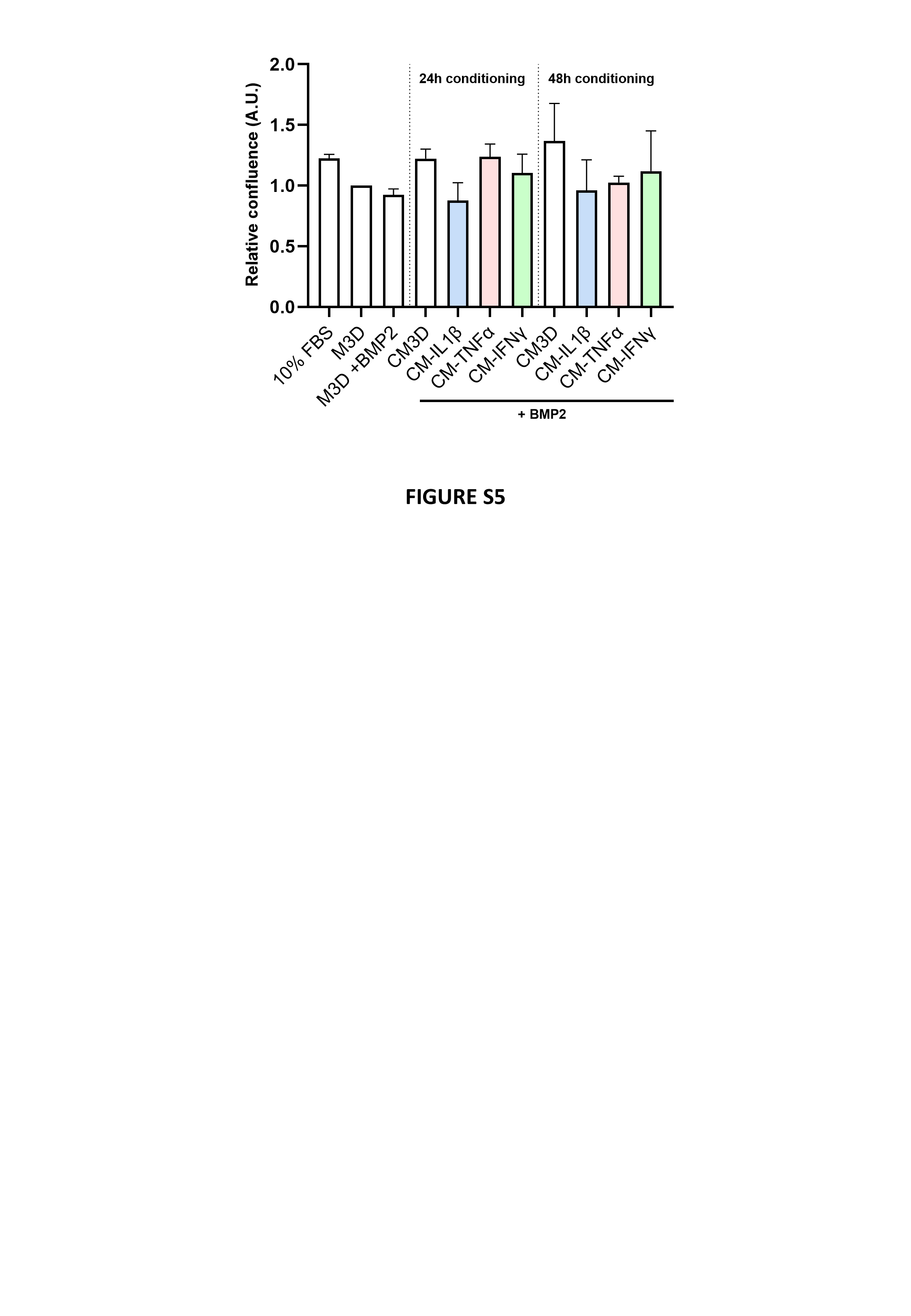

Supplement: Supplementary file 7 [file Image5.TIF]
