# Supplementary material for: Effect of pro-inflammatory cytokine priming and storage temperature of the mesenchymal stromal cell (MSC) secretome on equine articular chondrocytes
Source: Front Bioeng Biotechnol. 2023 Aug 31;11:1204737. doi: 10.3389/fbioe.2023.1204737 (PMC10502223; doi:10.3389/fbioe.2023.1204737)
Supplement: Supplementary file 6 [file DataSheet1.pdf]

Supplementary Material

Effect of pro-inflammatory cytokine priming and storage temperature of the mesenchymal stromal cell (MSC) secretome on equine articular chondrocytes

Manon Jammes, Romain Contentin, Fabrice Audigié, Frédéric Cassé, and Philippe Galéra*

*** Correspondence:** Philippe GALERA : philippe.galera@unicaen.fr

Supplementary table S1. Cytokines assessed in the MILLIPLEX® Equine Cytokine/Chemokine Magnetic Bead Panel (Millipore).

Cytokine	Significance
**CXCL1**	Chemokine (C-X-C motif) ligand 1
**CXCL8**	Chemokine (C-X-C motif) Ligand 8
**CXCL10**	Chemokine (C-X-C motif) ligand 10
**CX3CL1**	Chemokine (C-X3-C motif) ligand 1
**CCL2**	Chemokine (C-C motif) ligand 2
**CCL5**	Chemokine (C-C motif) ligand 5
**Eotaxin**	-
**IFN-γ**	Interferon γ
**IL-1α**	Interleukin 1α
**IL-1β**	Interleukin 1β
**IL-2**	Interleukin 2
**IL-4**	Interleukin 4
**IL-5**	Interleukin 5
**IL-6**	Interleukin 6
**IL-10**	Interleukin 10
**IL-12**	Interleukin 12
**IL-13**	Interleukin 13
**IL-17A**	Interleukin 17A
**IL-18**	Interleukin 18
**FGF-2**	Fibroblast growth factor 2
**G-CSF**	Granulocyte colony stimulating factor
**GM-CSF**	Granulocyte and macrophage colony stimulating factor
**TNF-α**	Tumor necrosis factor α

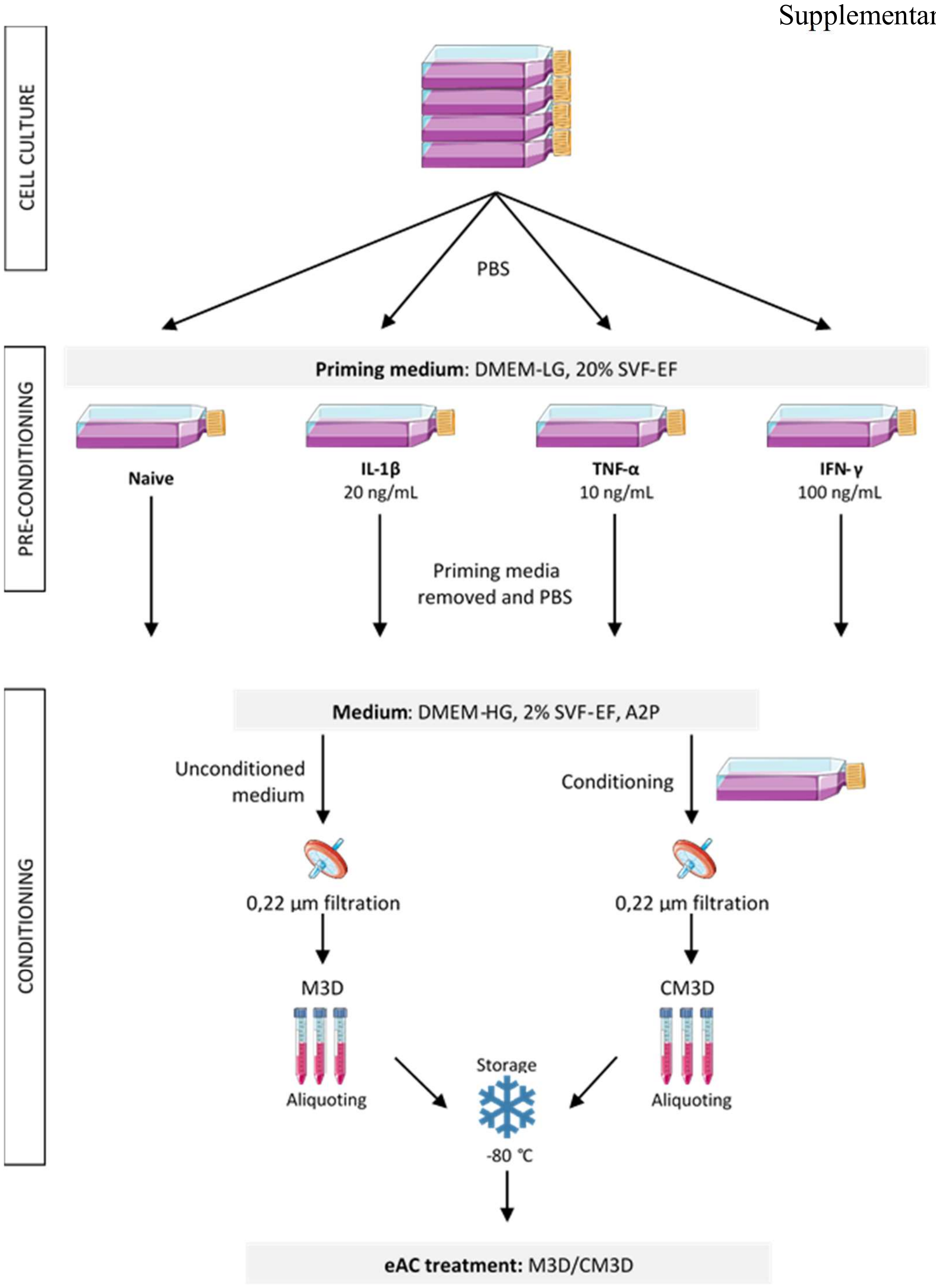

Supplementary figure S1. Experimental procedure for priming and preparation of conditioned media

When BM-MSCs reached 70% confluency, cells were rinsed twice with PBS and incubated at 37°C in a 5% CO2 atmosphere with preconditioning medium supplemented or not with equine IL-1β (10 and 20 ng/mL), TNF-α (10 and 20 ng/mL) or IFN-γ (50 and 100 ng/mL) for 6 or 24 h. Then, cells were rinsed twice with PBS and M3D medium was added for conditioning. In parallel, supplementary M3D was filtered at 0.22 µm, aliquoted and stored at -80°C to use as a control. BM-MSCs were incubated during 24 with M3D, and CM were collected, filtered at 0.22 µm, aliquoted and stored at -80 °C.

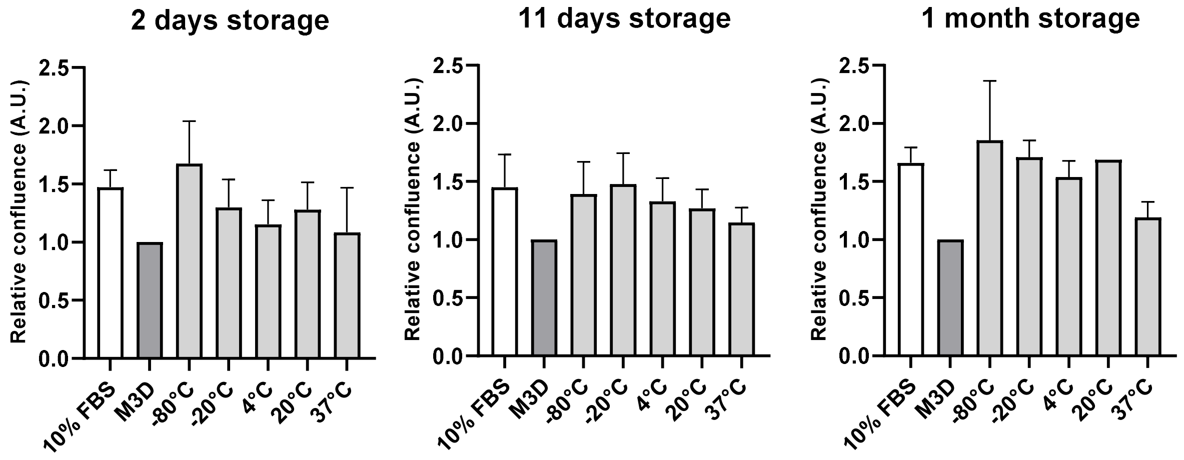

Supplementary figure S2. Effect of CM storage time and temperature on eAC proliferation.

CMs from BM-MSCs (P3) were harvested, filtered, aliquoted and stored at different temperatures for 2 days (n=3), 11 days (n=3) and 1 month (n=2). eACs (P2) were seeded in monolayer at 20 000 cells/cm². After 17 h, the culture medium was removed, cells were washed with PBS and treated with CMs. Cultures were monitored for 48 h with an Incucyte® live imaging system. Each condition was tested in triplicate and experiments were repeated 2 or 3 times. Cells were counted on 3 representative areas of each picture taken after 48 h of treatment and just after addition of CM. Relative cell confluence was determined from the ratio of the number of cells at 48 h to the number of cells at 0 h. Histograms show the impact of CM storage temperature – -80°C; -20°C; 4°C; 20°C; 37°C – for each timepoint. Mean values are represented as histograms ± standard deviation A.U.: arbitrary unit; BM-MSCs: bone marrow-mesenchymal stromal cells; CM: conditioned medium; eACs: equine articular chondrocytes; FBS: fetal bovine serum; M3D: control medium with 2% FBS; P2: passage 2; P3: passage 3.

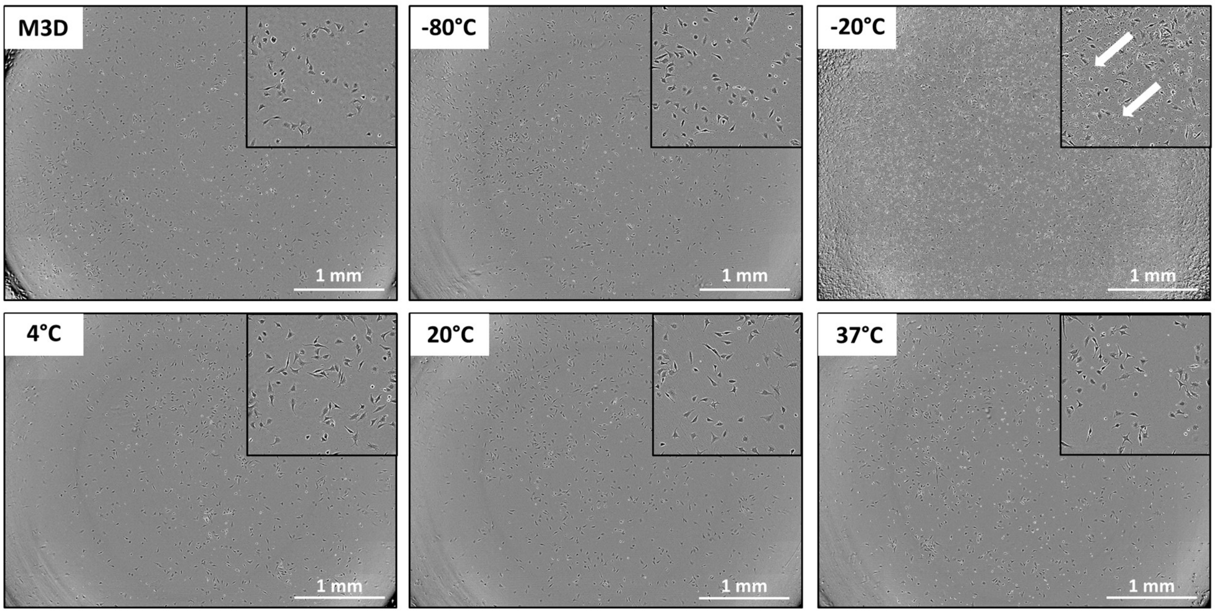

Supplementary figure S3. CM storage at -20°C leads to the formation of aggregates.

CM from BM-MSCs (P3) were harvested, filtered, aliquoted and stored at different temperatures. eACs (P2) were seeded in a 96-well plate at 20 000 cells/cm² for 17 h, and were then treated with CMs. Photomicrographs show eAC cultures 2 h after treatment (representative of two experiments). Arrows indicate aggregates. Scale bar, 1 mm. BM-MSCs: bone marrow-mesenchymal stromal cells; CM: conditioned medium; eACs: equine articular chondrocytes; M3D: control medium with 2% FBS; P2: passage 2; P3: passage 3.

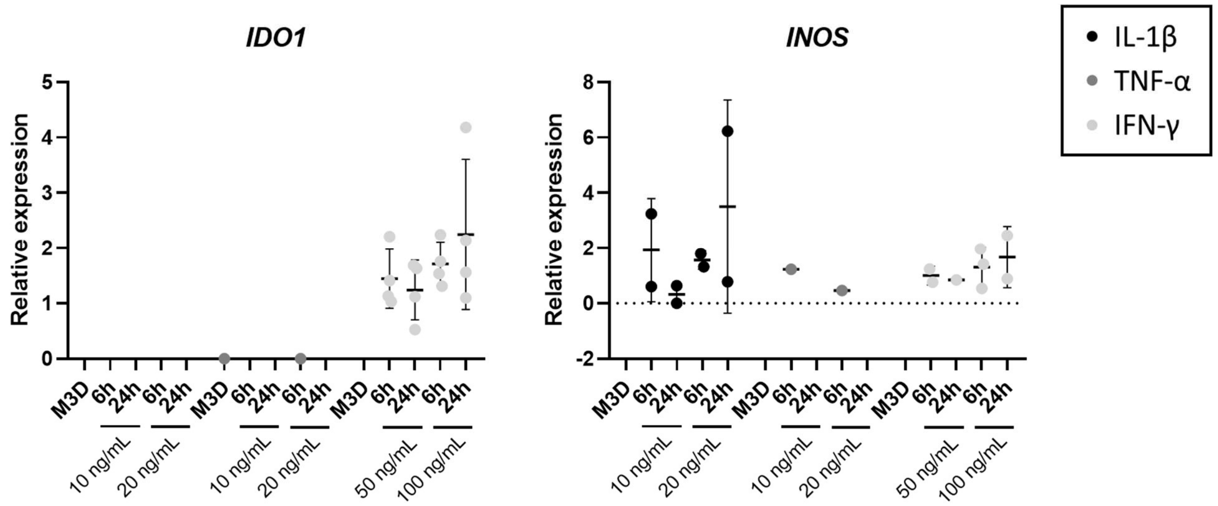

Supplementary figure S4. Effect of cytokine priming on the gene expression of *IDO1* and *INOS*.

At 50% confluency, MSC amplification medium with or without IL-1β (10 and 20 ng/mL), TNF-α (10 and 20 ng/mL) or IFN-γ (50 and 100 ng/mL) were added to BM-MSC cultures (P3) for 6 or 24 h. After two washes with PBS, media were replaced with M3D for 24 h. Then, media were removed, MSCs were washed twice again with PBS and stored at -80°C until RNA extraction (n=4). The gene expression of several immunomodulatory markers was assessed using RT-qPCRs, and normalized using the reference genes *β-ACTIN* and *PPIA*. Values are represented as box plots (median, quartiles, extreme values and mean (indicated with a "+"). BM-MSCs: bone marrow-mesenchymal stromal cells; M3D: unconditioned medium; CM: conditioned medium; eACs: equine articular chondrocytes; M3D: control medium with 2% FBS; P3: passage 3.

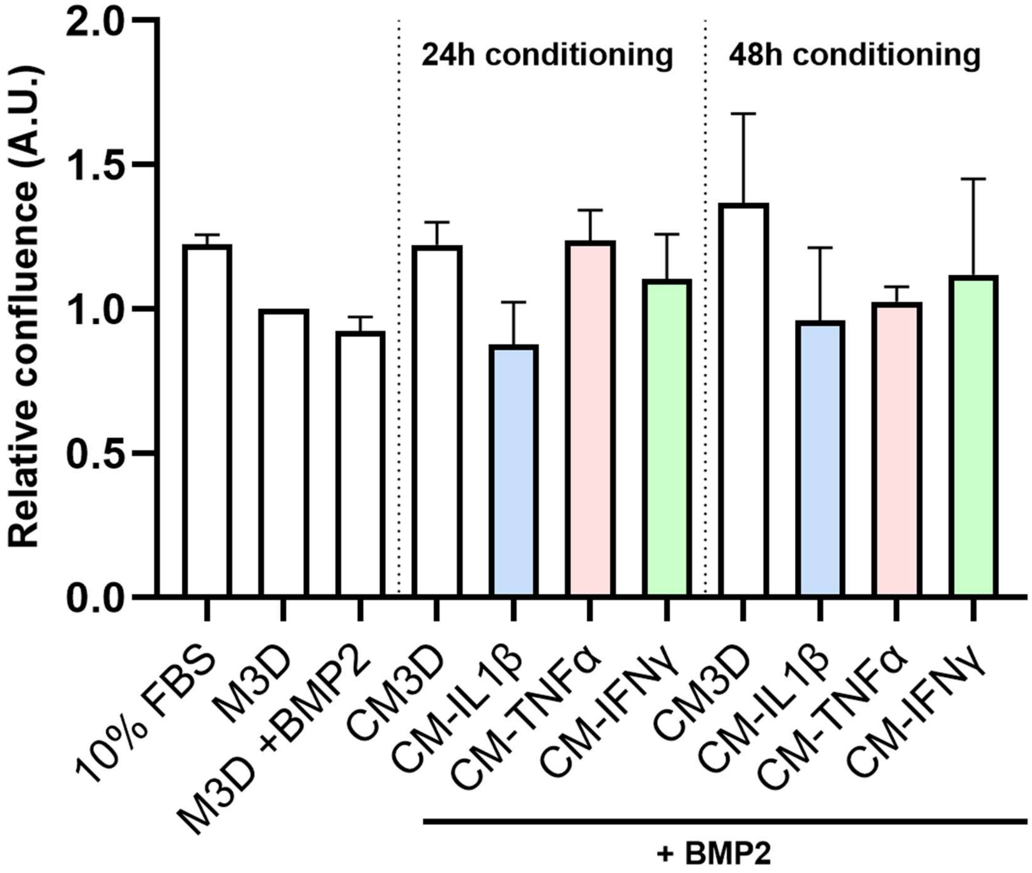

Supplementary figure S5. Priming with pro-inflammatory cytokines does not affect the eAC proliferation rate.

BM-MSCs (P3) were incubated with IL-1β (20 ng/mL), TNF-α (10 ng/mL) or IFN-γ (100 ng/mL) for 24 h. Then, cells were washed twice with PBS and M3D was added to cultures for 24 or 48 h. CMs from BM-MSCs were harvested, filtered, aliquoted and stored at -80°C. In parallel, eACs (P2) were seeded in monolayer at 20 000 cells/cm² in a 96-well plate. After 16 h, the medium was removed, cells were washed with PBS and treated with CMs, with or without BMP2 (50 ng/mL). Cultures were monitored for 48 h using an Incucyte® live imaging system. Each condition was tested in triplicate and experiments were repeated three times. Cells were counted on 3 representative areas of each image taken just after CM addition and after 48 h of treatment. Relative cell confluence was determined from the ratio of the number of cells 48 h to the number of cells at 0 h. Histograms show the impact of cytokine priming on the eAC proliferation rate obtained over all experiments (n=3). Mean values are represented as histograms ± standard deviation and were analyzed using the Mann-Whitney test to compare the difference with the M3D condition. BM-MSCs: bone marrow-mesenchymal stromal cells; A.U.: arbitrary unit; BMP2: bone morphogenetic protein 2; CM: conditioned medium; eACs: equine articular chondrocytes; FBS: fetal bovine serum; M3D: control medium with 2% FBS; P2: passage 2; P3: passage 3.

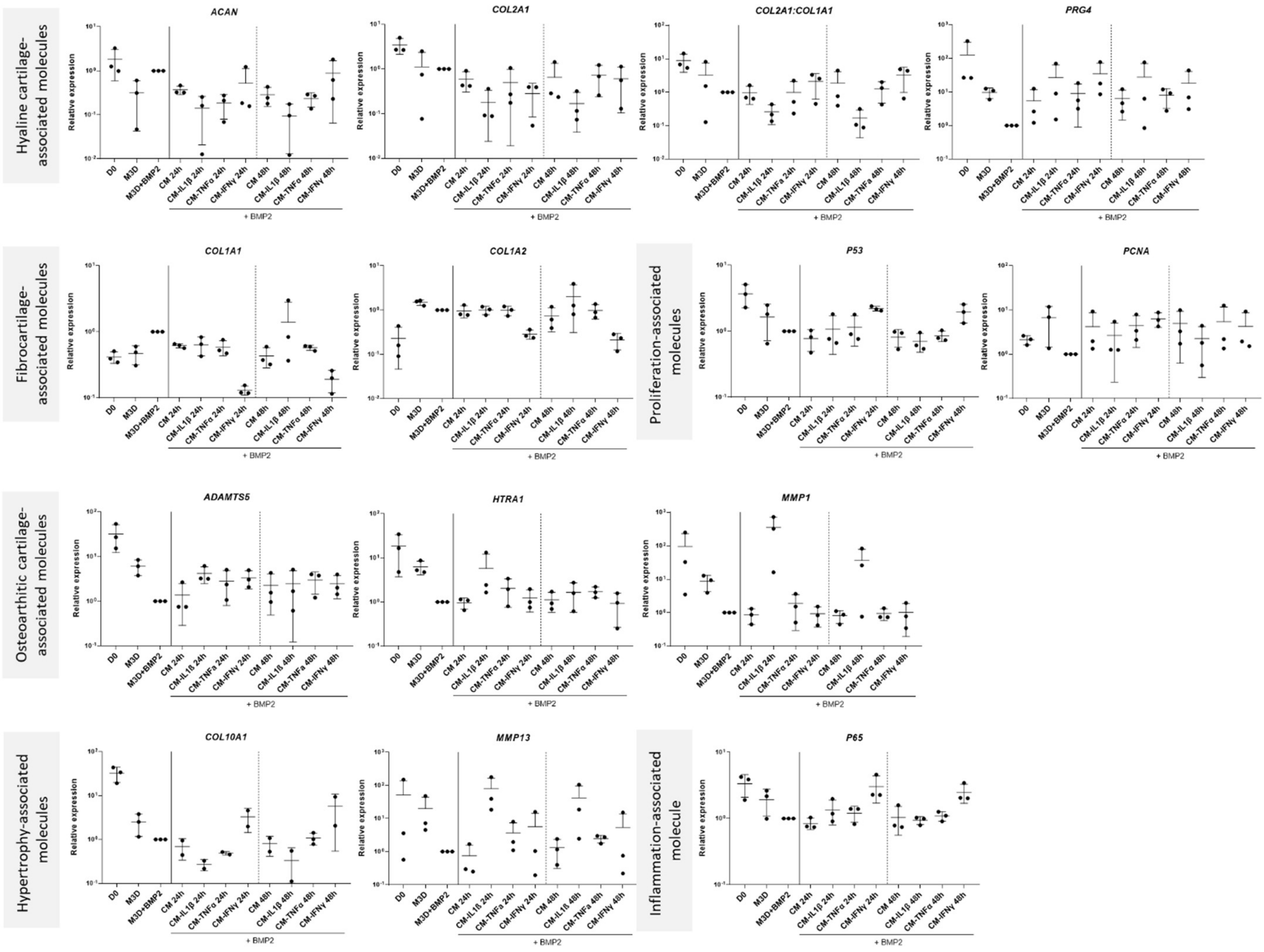

Supplementary figure S6. Primed CMs differentially modulate the gene expression of cartilage and OA-associated molecules in the presence of BMP2 in eACs.

BM-MSCs (P3) were incubated with IL-1β (20 ng/mL), TNF-α (10 ng/mL) or IFN-γ (100 ng/mL) for 24 h. Then, cells were rinsed twice with PBS and M3D was added to cultures for 24 or 48 h. CMs from BM-MSCs were harvested, filtered, aliquoted and stored at -80°C. In parallel, eACs (P2) were seeded in collagen sponges at 800 000 cells/sponge and, after 17 h, were treated with CMs and BMP2 (50 ng/mL) for 14 days. Then, sponges were harvested, washed twice with PBS and stored at -80 °C. Total RNA was collected from these cultures and RT-qPCRs were carried out on mRNAs to assess gene expression. The expression of target genes was normalized using the reference genes *β-ACTIN* and *PPIA*. The D0 condition corresponds to eACs cultured in monolayer until P2. Experiments were repeated with different strains of eACs and BM-MSCs (n=3). Mean values are represented as scatter dot plots ± standard deviation, and were analyzed using the Mann-Whitney test to compare the difference with the M3D+B condition. BM-MSCs: bone marrow-mesenchymal stromal cells; BMP2: Bone morphogenetic protein 2; CM: conditioned media; eACs: equine articular chondrocytes; M3D: control medium with 2% FBS; P2: passage 2; P3: passage 3; RT-qPCR: reverse transcription-quantitative polymerase chain reaction.
